# Deliver on Your Own: Disrespectful Maternity Care in rural Kenya

**DOI:** 10.1371/journal.pone.0214836

**Published:** 2020-01-07

**Authors:** Adelaide M. Lusambili, Violet Naanyu, Terrance J. Wade, Lindsay Mossman, Michaela Mantel, Rachel Pell, Angela Ngetich, Kennedy Mulama, Lucy Nyaga, Jerim Obure, Marleen Temmerman

**Affiliations:** 1 Centre for Excellence in Women and Child Health, Aga Khan University, Nairobi, East Africa, Kenya; 2 Department of Health Sciences, Brock University, St Catharines, Ontario, Canada; 3 Aga Khan Foundation, Canada Ottawa, ON, Canada; 4 Aga Khan Foundation, East Africa, Nairobi, Kenya; Liverpool School of Tropical Medicine, UNITED KINGDOM

## Abstract

**Background:**

Under the Free Maternity Policy (FMP), Kenya has witnessed an increase in health facility deliveries rather than home deliveries with Traditional Birth Attendants (TBA) resulting in improved maternal and neonatal outcomes. Despite these gains, maternal and infant mortality and morbidity rates in Kenya remain unacceptably high indicating that more needs to be done.

**Aim:**

Using data from the Access to Quality Care through Extending and Strengthening Health Systems (AQCESS) project’s qualitative gender assessment, this paper examines women’s experience of disrespectful care during pregnancy, labour, and delivery. The goal is to promote an improved understanding of the actual care conditions to inform the development of interventions that can lift the standard of care, increase maternity facility use, and improve health outcomes for both women and newborns.

**Methodology:**

We conducted sixteen focus group discussions (FGDs), two each for adolescent females, adult females, adult males, and community health committee members. As well, twenty-four key Informants interviews (KII) were also conducted including religious leaders, and persons from local government representatives, Ministry of Health (MOH), and local women’s organizations. Data were captured through audio recordings and reflective field notes.

**Research site:**

Kisii and Kilifi Counties in Kenya.

**Findings:**

Findings show nursing and medical care during labour and delivery were at times disrespectful, humiliating, uncompassionate, neglectful, or abusive. In both counties, male health workers were preferred by women giving birth, as they were perceived as more friendly and sensitive. Adolescent females were more likely to report abuse during maternity care while women with disabled children reported being stigmatized. Structural barriers related to transportation and available resources at facilities associated with disrespectful care were identified.

**Conclusions:**

A focus on quality and compassionate care as well as more facility resources will lead to increased, successful, and sustainable use of facility care. Interpreting these results within a systems perspective, Kenya needs to implement, enforce, and monitor quality of care guidelines for pregnancy and delivery including respectful maternity care of pregnant women. To ensure these procedures are enforced, measurable benchmarks for maternity care need to be established, and hospitals need to be regularly monitored to ensure these benchmarks are achieved.

## Introduction

Few health interventions have greater potential impact on the overall health of society than good quality, facility-based care to women while pregnant and during and after childbirth. One of the factors that has been shown to affect utilization of facility-based, maternal health care services is the experience of disrespectful care received by women. Respectful Maternity Care (RMC) is a universal human right, which entails respect for the individual and their beliefs, traditions, and culture. It encompasses a continuity of care, the right to information and privacy, good communication between client and provider, and use of evidence-based practices’ [1–3]. Respectful care also includes the right to receive care free from harassment, humiliation, discrimination, neglect, and abuse [2].

Over the past two decades, the RMC movement has gained considerable attention worldwide as a basic right of women throughout pregnancy, labour, and delivery. The World Health Organization (WHO) recognizes the principle of respectful care as a major factor for increasing the use of pregnancy and maternity healthcare services resulting in better maternal and neonatal outcomes[2–4]. Research has shown that women who perceive receiving substandard and disrespectful care during childbirth are far less likely to seek future facilty-based birth care which places them and their newborns at risk [4–6]. Moreover, women with negative experiences may also discourage others from seeking facility-based care [7]. Moreover, vulnerable women in lower social economic positions are at a greater risk to experience disrespectful care despite existing professional standards and documented policies with respect to delivery of care, even among High Income Countries (HICs) where legal consequences for violations in providing care exist and patients are aware of their rights[8]. These findings support existing research from LMICs that shows that women about to give birth, especially poor and rural women, are more likely to report being neglected, humiliated, and often subjected to verbal and, at times, physical abuse violating the principles of respectful and compassionate maternity care [9–12]. Some explanations for disrespectful care include inadequate staffing, outdated and substandard equipment and resources, and a lack of explicit ethical standards, training, and enforcement for professional care. For example, lacking adequate basic medical necessities to ensure the appropriate delivery of respectful and suitable health care makes it more difficult for healthcare workers to do their jobs increasing the likelihood that they will engage in substandard and disrespectful care such as routinely ignoring patient requests. Other research indicates that gender inequality and unequal power distributions may also act as a barrier to respectful maternity care [13, 14]. Moreover, women from low-resource, poor and rural settings are more likely to experience gender inequalities and to be discriminated against by health care workers due to their low status in the society [15, 16]. As such, the unequal distribution of power between men and women, and women’s lack of autonomy in decision making at the household level among women who are poor or from more rural areas exposes them to greater term health risks–with enormous social and economic consequences[17].

In Kenya, under the Free Maternity Policy (FMP) established in 2013, an increasing number of women have elected to give birth in maternity care facilities rather than at home with traditional birth attendants (TBA)[18, 19] [18,19]. Despite these gains, infant mortality and morbidity rates in Kenya remain stubbornly high and not all pregnant women may be willing to attend facility care services. Emerging anecdotal evidence suggests that this reluctance to utilize facility maternity care may be, in part, that some pregnant women seeking maternity care report neglect and abuse[20] owever, there exists little research evidence to corroborate these stories in Kenya. In an attempt to begin to address this gap in the literature in Kenya, this study analyzed data from the qualitative gender assessment of the Access to Quality Care through Extending and Strengthening Health Systems (AQCESS) project that examined the maternity care experiences among Kenyan women in two dissimilar rural settings. The Gender Assessments Project was conducted by Aga Khan Foundation Canada (AKFC) and implemented by agencies of the Aga Khan Development Network (AKDN) with financial support from the Government of Canada, through Global Affairs Canada (GAC) and the Aga Khan Foundation Canada (AKFC). The overall aim of the Gender Assessment Study was to begin to understand the gendered dimensions of access to and control over resources, decision-making, social norms, and perceptions and practices related to access and use of Maternal and New Child Health Services in Kisii and Kilifi counties in Kenya. This paper builds on the learnings of that assessment and reanalyzes the data to focus specifically on the reported experiences by participants related to disrespectful maternity care

### Study ethics approval

Before commencing the study, the Aga Khan University’s (AKU’s) Ethics Review Board and the National Commission for Science, Technology, and Innovation (NACOSTI) approved the study (ref NACOSTI p/17/1475/16146). In addition, approval to commence data collection was also provided by the respective County offices at both sites.

### Methods

The data came from two target locations for the AQCESS project in Kenya’s rural Kilifi and Kisii Counties.

#### Kilifi county

Kaloleni and Rabai sub-counties, with a combined population of 304,778 were selected as target sites in Kilifi County and are among the poorest areas in Kenya with approximately 70% of the population living below the poverty [21–23]. Some of the risk factors that lead to poor health outcomes in this context include poor hygiene and sanitation and low literacy levels, particularly among women 22, 23]. During the last decade, maternal mortality rates increased in Kilifi with 448 deaths per 100,000 live births estimated for 2016 compared to 414 per 100,000 live births in 2004 despite the 2013 implementation of the Kenya Free Maternal Policy. Kilifi County also has one of the highest under 5 mortality rates across Kenya with 87 deaths per 1,000 live births [22, 23]. Finally, 21% of adolescents in Kilifi had pregnancies in 2016 which was slightly above the national average [22].

The two sub-counties are served by 40 health facilities of which half are public/government health facilities (16 dispensaries, one health centre, two sub-county hospitals, and one military health centre), and the rest being faith-based, NGO, or privately owned. The health system in the two sub-counties faces numerous structural challenges including limited human healthcare resources, poor access to health services due to geographic and transportation barriers, and limited healthcare infrastructure [21]. The physician and nurse to population ratio for Kilifi County is about 1 to 48,000 and 1 to 8,594 respectively, which is considerably lower than the national averages of 1 to 36,000 and 1 to 5,000[22, 24].

#### Kisii county

The sub-county Bomachoge Borabu, where this research was conducted, is one of nine constituencies in Kisii County. In 2009, the population was 129,617, with an estimated population growth rate of 3% in 2016 [23]. 21% women are of reproductive age and there are approximately 5,055 deliveries per year with a crude birth rate of 26/1000 population per year [25]. A rural county where most people are engaged in subsistence farming, the poverty level is 51%, which is higher than the national poverty index (44%) but lower than Kilifi County[25]. The percentage of teenage women 15 to 19 years of age who have begun childbearing is 22%, similar to Kilifi and above the national average. The sub-county is serviced by 12 health facilities including one sub-county hospital, five government dispensaries, five private clinics and one faith-based health centre. The doctor and nurse to population ratios in 2014 were about 1:52,000 and 1:3,900 respectively.

### Study sample participants

To ensure that the data are representative within and across the two communities and their respective health systems, participants were selected through a recruitment process involving support from the AQCESS project implementation teams and local community leadership. The recruitment process was successfully implemented by a combination of health facility liaisons and community health outreach workers. First, the researchers conducted 24 Key Informant Interviews (KIIs) distributed equally across the two sites. KIIs were purposively sampled with the support of the Kisii and Kilifi AQCESS project implementation teams, identifying potential respondents with key information related to the assessment areas of inquiry. They included health care providers, community health care workers, religious leaders, local government representatives, Ministry of Health representatives, and representatives of local women’s organizations.

Second, 16 Focus Group Discussions (FGDs), 8 in each county, were facilitated to include a broad representation of community members and community health committee members. FGDs were stratified to include 2 each for adult males; adult females aged 20–49, and adolescent females ages 16–19. The separate adolescent female FGDs were conducted to ensure that the specific experiences among female adolescents were captured. To allow for full and unrestricted participation, FGD participants were also purposively recruited to ensure geographic representation so that 50% of the FGDs were with participants living less than 5km from a facility and 50% of FGDs were with participants located more than 5kms from a facility. This criterion is based on past evidence indicating that distance plays a major role in decision-making around the use of services about whether to seek care and to actually access care. The final two FGDs for each county included both male and female members from the community health committees (CHCs).

#### Interview protocol

All KIIs and FGDs were conducted in April 2017 by AQCESS trained research personnel at venues convenient to the participants. The field teams conducting the sessions received training on the interview protocol and tools, and on gender sensitive research and ethics. Interview guides for both participants and interviewers were available in relevant languages including English, Swahili, and local dialects (see S1 Appendix and S2 Appendix). Informed written consent was provided by all adult participants. For adolescent females participating in the study, both their assent and the consent from an adult including a parent or guardian. A community health volunteer was approached to give consent where there was no parent or guardian and if they lived in the same community as the adolescent. All adolescent females involved in this study had at least one child and were 16 years of age or older. During the consent, process researchers explained the purpose of the study, the potential risks, that participation was voluntary, and participants could withdraw at any time.

For the FGDs, qualified moderators conducted the discussions in Swahili, local dialects, or English, as appropriate. All FGDs and KIIs took no more than 2 hours and all sessions were recorded with the permission of respondents. Trained individuals conversant in English, Kiswahili and the local dialects, transcribed audio recordings.

#### Data management and analysis

Audio recordings needing translation from Kiswahili or local dialects to English were translated for analysis. The process entailed listening to the recordings and typing the content and translating into English as required. A transcription supervisor checked all submitted transcripts and translations before they were stored for coding. Transcribed data were coded, encrypted, and saved securely in accordance with the AKU’s Ethics and Data Protection Act.

All qualitative data were analyzed using a continuous iterative process[26]. Each transcript was coded independently by two analysts and reviewed by a study investigator who was the chief data analyst. Where coding discrepancies occurred, at least two analysts re-examined the transcripts and discussed all possible meanings associated with the text in question until agreement was achieved. The current analysis relied on the users’ own report of their experience of disrespectful maternity care services. The coding of key themes is presented in Table 1 below.

**Table 1:** Codes, categories and themes of disrespectful care.

CODES	CATEGORIES	THEMES
Left alone in pain; Health workers refused to assist; Delivered on my own; Staff read newspapers; Woman waited for long hours without help; Health workers ignored woman; health workers told woman to deliver on her own.	Lack of professionalism; Lack of compassion; Lack of respect and dignity.	**1. Neglectful Care**
Young woman harassed; Young woman isolated; Doctors harsh to young woman; Young woman forced to share information about the child’s father; Young woman quarreled; Young woman humiliated while in pain; Young woman slapped; Young woman contemplated killing her baby; Young woman called prostitute.	Mistreatment; Age based discrimination; Physical and verbal abuse; Women’s dignity; Women’s basic rights.	**2. Adolescent women get the brunt.**
Slapped by female nurse; Delivered by a male nurse; Male nurse angry with female nurse for not helping; Male nurse was very kind; male nurse was helpful; I prefer male nurse; Male nurse preferred; Female nurse proud; Female nurse has no mercy; I fear female nurse; abused by female nurse for having child with disabilities. Called prostitute for having child with disabilities.	Differential gendered experience of care; Treatment of women by women health workers causes fear; Inappropriate obstetrics; Discrimination, equality of care.	**3. Patients’ differential gender experience of health workers**
The hospital was far; wheeled on a wheelbarrow and baby died. Staff were few; Carried a Jerry can of water on my head to the hospital while in labor; Delivered on the floor; Few beds in the ward; Few wards; Few theatres.	Resources; Lack of water; Inadequate facilities; Low staffing; Physical environment.	**4. Structural factors as a barrier to respectful maternity care**

## Findings

Pregnant women across both sites seeking antenatal care as well as women in labour, during delivery, and after delivery report that some health care workers in maternity care facilities were, at times, disrespectful, demeaning, humiliating, verbally or physically abusive, dismissive, and neglectful of patient-reported pain and suffering. Although our results illustrate that women of all ages experienced these types of inappropriate care and neglect and abuse, younger women, especially adolescents, were more likely to report verbal and physical abuse. As well, both male and female focus group participants suggested that female health workers were more likely to demonstrate abusive behaviors and, as a result, many participants indicated that they preferred to be tended to by male health workers. However, some Muslim participants reacted negatively toward being attended to by male health care workers. Below, we present the specific themes that emerged from the data analysis and provide vignettes to illustrate and support them.

### 1. Neglectful care

Data from both research sites provided evidence in which patients’ basic rights to care were violated and where healthcare workers were inattentive or ignored patients’ requests for assistance. For example, speaking of her own personal experience with maternity healthcare workers, one of the adult female FGD participants reported that some of the health workers refused timely help to women at the time of delivery.

…My experience with my first-born delivery was not good with a woman nurse. I told her I was in pain, and she abused me saying I should ‘stop my nonsense and wait to give birth in the morning.’ Soon after, a male nurse came and assisted me [to] give birth. He even quarreled [with the female nurse], asking her why she was treating me that way.(Adult Female FGD, Kisii).

In the following, a mother described her experience while accompanying her relative and the treatment her cousin received convinced her never again to go back to a public health care facility.

Haaa, I can’t, I better go to the private clinic … With those mockery and abuses I can’t. Like when we went at night with my cousin. There was a male doctor who we think was on drugs—chewing miraa, was sitting outside, maybe he was taking bhang. He told the girl ‘it’s not you [who can] tell us what to do!’ When the pain was too much, she went to the bed on her own, and the man came wore glove inserted his fingers and literally tore the lady… (Adolescent Female FGD, Kisii).

A key informant interview with a local politician highlights this pattern of neglectful and uncompassionate approach to care at the facility.

…I have witnessed a case at the hospital where there was a woman who came for the first time and she was told she would not be attended to until she brings her husband … she went and never came back (Women representative KII- Kisii).

In the following example, a participant in an adult male FGD speaks of his experience with staff while accompanying his relative to the hospital.

I took my sister in-law to hospital, and after assessing her, they left her alone in bed. She called for assistance, but they said, ‘It’s not yet time.’ She pushed and delivered on her own, that’s when the health workers came running wiping the child…at times they can beat you because you gave birth on the floor, as if the mistake is yours. Yet they don’t respond when called (Male FGD, Kisii).

This neglectful behavior by healthcare staff was further demonstrated by a general inattentiveness and disregard toward patients. Some healthcare staff who neglected patients were reading newspapers. As well, healthcare workers sometimes left the facility altogether, going for long lunch “hours” and leaving patients for hours on end, waiting to be attended to:

I can add that medical providers are very… abusive especially for young ladies and adolescents. Some medical staff decide to go for early lunch. Some read newspapers while patients are waiting for services (Male FGD, Kisii).

It happened that since she declined going to theatre the health workers ignored her and told her to deliver on her own, but God intervened [by chance]. So the nurse’s attitude, Ignorance could be one of the things that contributes to this. (Male FGD, Kisii)

The above vignettes demonstrate some of the ways in which women were treated while under the care of health care workers. These vignettes also demonstrate a lack of professionalism and compassion among some health workers.

### 2. Adolescent women get the brunt

While some of the adult women reported that they experienced different kinds of incivility and poor care, the adolescent women focus groups reported that they were more likely to experience verbal and physical abuse by healthcare workers in both Kisii and Kilifi. A focus group discussion among adolescent women in Kisii confirmed that some doctors also share this prejudice. In this case, a doctor used harsh and humiliating language when speaking to a young pregnant women during the delivery process.

… During the pain, they [doctors] abuse you and tell you to deliver. Yet you don’t know anything. They tell you to push, and you don’t know how.” (Female Adolescent FGD, Kisii).

…We (adolescents) are attended to, but harshly. For example, me, I’m young and they told me, ‘Young as you are, you went opening your legs to men.’ (Female Adolescent FGD, Kisii)

Some adolescent women reported being slapped and disrespectfully interrogated about the father of the child during the labour process. One was so upset by abusive nature of some staff, she contemplated killing the child.

She [the nurse] was slapping me. I said, ‘I’ll kill the baby and give birth to a dead baby,’ but the lady who escorted me went and called for a male doctor, who assisted me… (Female Adolescent FGD, Kilifi)

… Some doctors are harsh because you’re a girl, during the pain they abuse you and tell you…’Young girl, when you were loitering looking for that pregnancy, were we there?’Imagine! Instead of helping you… (Female Adolescent FGD, Kisii).

… They ask you questions, ‘When did you get pregnant? Who gave you this pregnancy?’ You don’t know what to answer them. If you don’t answer, they leave you there unattended, and say ‘Till you answer …’ (Female Adolescent FGD, Kisii).

As the following discussion from one male FGD particpant illustrates, this kind of poor treatment appears to stem from social disapproval, leading to disrespect for adolescent girls who become pregnant.

*Respondent*: Our young wives, usually they encounter a lot of problems.

*Moderator*: Oh, young wives? Ha!

*Respondent*: Yes, the young ones. You know us. We have been, ah, so

(laughter) … when they go there, and especially because they are young, they complain that they are harassed and asked: ‘You! As young as you are you …,’ [and] such like harassment.

Moderator: Why such harassment?

*Resondent*: Because they are young and are already pregnant. And what are they expected to do, and it has already happened?

*oderator*: Okay. So how is she harassed? (Group reaction)

*Respondent*: She gets uncomfortable.

*Respondent*: They begin to think ‘this young girl has already conceived,’ so when she goes there to be served, she feels out of place and fearful, hence feel she has not been served well. She feels like she has been isolated (Male FGD, Kilifi).

While some women generally suffer abuse from maternity facility caregivers, levels of physical abuse were reported to be greater for younger women during the actual delivery.

*Respondent*: Actually, it is during delivery that there are usually a lot of difficulties.

*Moderator*: Kindly mention all.

*Respondent*: Some of [the women] are slapped, and that story became a talk of the town some time back, yeah. Some of the doctors [show] that behavior. (Okay). ‘You did it willingly, and you want to cause us trouble now.’ You see, that’s bad to tell somebody. She might decline to come back again, when she is pregnant in the future, and will prefer to home delivery. … Yeah, there is such a one [who slaps the women] here, only that I cannot disclose, but they are there. Very short-tempered, even tells you not to come back to that hospital (Male FGD, Kisii).

Poor treatment of young women was also reported in some of the Key Informant Interviews. In the following vignettes, two Ministry of Health (MOH) representatives observe:

…the way they [staff] handle these mothers, somebody may harass the mothers and next time… or even when she goes back she will go with a bad picture and says,’ I cannot go back to that facility, they do not handle people properly, they call us with very abusive words …. (MOH representative KII–Kisii).

…“I guess once again I would say the adolescents are quite disadvantaged because even the stigma is within the medical care providers, so in terms of the MCH, it is very difficult for the adolescent mothers because they need to seek health care, yet providers’ attitude is the worst when dealing with adolescent mothers.” (MOH representative -Kisii).

These vignettes illustrate the differential experience of maternity care among adolescents with respect to disrespectful and abusive health care. Moreover, the similarity among the vignettes from both the FGDs and key informants show a consensus regarding the greater level of disrespectful care that these adolescent mothers must tolerate.

### 3. Patients’ differential gender experience of health workers

The data showed that some women in Kenyan maternity care facilities generally found that female healthcare workers were more likely to be disrespectful and abusive compared to male healthcare workers. Some participants in the focus groups expressed their preference for male healthcare workers who they indicated treated them better and were more willing to help when asked. Adolescent women, as a group, were the most vocal in expressing their negative opinion of female staff and their preference for male health care workers.

…especially ladies, they are so harsh; they think they dropped from heaven [special than everyone] and us were collected. Men are better; they can tell you to push while assisting you. But females will slap you, yelling at you to open your legs while shouting ‘I want to see the child!’ with abuses. She tells [me] ‘I am waiting to hold the child! (Female Adolescent FGD, Kisii).

… Especially female doctors are the worst. Men don’t have problems. If you come with a Range Rover or a [Mercedes] Benz, they will wheel you to the ward. But if brought with a wheelbarrow, you’ll be told to move from here to there—they don’t mind the pain you have. You’ll be locked inside a room, and be told to yell there. I was locked [in] and told go for long call there (Female Adolescent FGD, Kisii).

While participants interviewed for this study generally reported the preference of male attendants as they provided more respectful care, Muslim patients’ desire for male caregivers was tempered by cultural and religious beliefs. One Muslim participant in the adult male focus group reported how his wife resisted being assisted by a male worker.

I think the most interesting thing is that the women prefer to be served by male health workers rather that the females…Yes that is the truth but not so for us as Islamic. My wife declined totally to be assisted by a male nurse. Even during the clinic, she forbids the male nurse to even touch her when she is to be injected…Actually, it happened that the baby’s head was already out before she could even be assisted. The only person that was available was a male nurse. You know in our religion we prefer not to show the nakedness of a woman—it is sacred… (Male FGD, Kilifi).

Female healthcare workers were reported to be verbally and physically abusive resulting in patients generally preferring to be attended by male health workers.

*Moderator*: So, you are saying that women prefer the male health workers to the female?

*Respondents*: Yes! (Chorus answer)

*Respondent*: Yes, because the male nurse has a patient heart. He will even try to console the mother, unlike the female nurse, who can inflict slaps. Then she goes about her businesses, leaving the mother behind without even caring (Female Adult FGD, Kisii).

Participants reported that women who gave birth to children with disabilities were likely to be humiliated by some female staff who blamed the mother for the child’s disability.

They are women and maybe they’ve also given birth. They should know the pain they went through … If you give birth to a disabled child, they ask you when coming to the clinic, ‘Were you moving with men when pregnant?’ Or, ‘Your man did fix you well’ (hakuingisha [penetrate] vizuri [good or satisfactory]) so the child didn’t reproduce properly. The man didn’t have energy.’ (Female Adolescent FGD, Kisii).

…female health workers have contempt. When we went with my cousin, who is also young, we brought a disabled child. Another lady nurse abused us, till we also abused her back. . . . We looked for another doctor, the nurse abused her saying: ‘She was sleeping with men while pregnant, that’s why she gave birth to a disabled child!,’ and we reported the case to the senior doctor…We don’t know if she was reprimanded. My cousin wanted to kill the child, saying she is abused because of her child status. We later took the child to my grandmum, who took care of the child (Female Adolescent FGD, Kisii).

These vignettes illustrate some of the gendered differences among healthcare workers as reported by patients. These vignettes also illustrate some of the attitudes and beliefs associated with giving birth to a child with disabilities, an area that has not been addressed in research or policy in the Kenyan context.

### 4. Structural factors as a barrier to respectful maternity care

Our analysis confirms that, in both Kisii and Kilifi, public prenatal and maternity health care failed to treat some women with dignity and respect. However, respectful care is also contingent on the availability of structural resources to support women on their journey through maternity. Findings from this study reveal that some maternity health centres may lack basic resources and supplies such as water, beds, or readily fueled vehicles and ambulances to transport patients that are referred to larger facilities (see S3 and S4 Appendices). For example, participants reported that in some cases women carried water along with them to the hospitals for their delivery.

…You can find a woman who is in labour carrying a jerrican of water on her head going to the hospital. Simply because she knows there is no water at the hospital… (Male FGD, Kilifi).

..I saw a woman groaning in pain she had come to deliver and there was no water. Usually there is scarcity of water in this area. It happened that that day the care provider present was not supposed to be on duty that night. So it happened as I was talking with her that is when that mother came in but she had to be send elsewhere because the hospital was not functioning to the lack of water… (Male FGD, Kilifi).

Spaces and beds were not adequate to meet the demand of the number of pregnant women seeking care at some facilities.

The beds in the labour ward should be added. The wards are also small. Some women are usually waiting to give birth while lying down on the floor because the beds are occupied. When I was delivering, I gave birth while lying on the floor because the beds were occupied and there was nowhere to deliver. I knelt and the baby came… (Female Adult FGD, Kilifi).

The lack of availability of beds for mothers may be discouraging the uptake of maternity services. For some women, other structural barriers including poor road systems and inadequate means of transportation and lack of available healthcare staff hindered their access and use of the facilities. Participants in the male FGDs in both Kisii and Kilifi reported the difficulties that some women face in getting to the facilities which can have an extremely tragic effect on maternal outcomes.

…I come from a place called Ibencho, roads are in bad condition. There is a woman who wanted to deliver and was carried using a bed and because of the distance, she died before reaching the hospital…this was less than five years ago. From Ibencho, people are only carried using beds or wheelbarrows to Sengera …Things are not different with Riokindo.” (Male FGD, Kisii)

… I have always witnessed women suffering and having a rough time in accessing the facilities due to long distances that they have to cover. And if it is a must they get to hospital the only available means of transport is the motor bikes. So you can imagine a pregnant mother being rode on a motorbike, it is usually a hard task. This is a challenge. So that is what I have been able to witness also sometimes it happens that some due to that they end up having complications and some even may die before getting to the health facility. This I have witnessed many times and secondly, when they get to the hospital you find that midwives are not available (Male FGD, Kilifi)

These vignettes demonstrate that on a woman’s journey through pregnancy and delivery, there are many structural barriers that she must navigate both at the micro and the macro levels. Disrespectful care is not confined solely to how healthcare workers treat women but must consider additional factors such the availability of resources required to provide access to and the availability of respectful and appropriate care.

## Discussions and policy implications

From these results, it is clear that women experience disrespectful maternity care by some healthcare workers, particularly by female staff. The strong support for the presence of disrespectful maternal care throughout the maternity process appears to be even greater among women who are poor, young, distant, or have children with disabilities. Consistent with research in other LMICs [6–8, 11, 17, 18] examining disrespectful maternity care, there is no reason to believe that the results would not be generalizable to other Kenyan communities. The widespread practice of disrespectful care has the potential to undermine the efficacy and reputation of the entire Kenyan public maternity health care system and the longer-term success of the recently implemented Free Maternity Policy in reducing maternal and child mortality.

Our findings raise issues around various aspects of delivering acceptable and respectful care including social cultural norms, the gendered nature of maternity care, the stigma around poverty, age, pregnancy, and disabilities, and the structural barriers and inadequacy of resources for maternal care. Moreover, the consistency of these reports with findings from other low and middle income countries [12–15], especially the treatment of adolescent women, shows that these are not isolated experiences suggesting there are social cultural undercurrents that guide treatment towards these more marginalized groups. These results demonstrate an urgent need to address these social and cultural issues to ensure that care provided under the Kenyan Free Maternal Policy is safe and satisfactory care to reduce infant and maternal mortality.

To accomplish this, rather than looking at the disrespectful and neglectful nature of some health workers in isolation, a systems perspective theoretical approach can provide insight on how to address mishaps within the larger social, cultural, gendered, and structural context in the delivery of maternity care. Within a systems approach, while it is the responsibility of all individual healthcare workers to strive to promote respectful maternity care, placing the onus solely on individuals in isolation of the larger social, cultural, and structural context will not solve the inadequate services pregnant women receive. As illustrated in Table 2 below, a systems perspective approach posits that behaviors are part of a larger system including all the structures that support that system beyond patients and health care workers at individual facilities. In this case the need to consider policy makers and leaders, medical boards, national governments, local governments, international regulators [WHO), community health committees, and others. That is, to ensure that women receive timely and respectful health care at all times, all health system actors must be engaged and coordinated to promote functional environment where safe, compassionate, and respectful care is the expected norm.

**Table 2:** Below provides a summary of our findings, suggestions and possible future research from a system perspective.

What is the problem?	Who is responsible?	Proposed interventions	Future research
1. Verbal and physical abuse of pregnant women attending Facility care. 2. Age: Abuse and humiliation of young pregnant women seeking maternity care. 3. Gender: Female health workers likely to abuse pregnant mothers 4. Gender: Mother-attending services with a disabled child likely to be stigmatized and humiliated. 5. Health system: Inadequate facilities e.g water at the Facility level to support pregnant women during delivery	Using a system theory perspective: 1. World Health Organization (WHO) must set standards, follow up on enforcement, demand audits and monitor progress. .2 The government of Kenya (GOK) through the Ministry of Health must enact and enforce standards of operation. 3. Following the devolved government in Kenya, the Local county governments must provide adequate facilities for pregnant women, initiate relevant training on respectfiful maternity care and increase incentive to staff. 4. Medical Boards: Nursing Council of Kenya 5. Legislators: Must formulate policy and ensure that it is implemented and enforced. 6. Hospital Boards: Mandated with managing health facility must ensure that staff are well trained and standard operating procedures are followed included patients right to respectful care.	1. Training of clinical and non-clinical staff to combat negative attitudes on age and stigma around disabilities 2. Enforcing standards of care with clear benchmarks 3. Mandatory Continued Medical Training on respectful maternity care. 4. Engaging all health systems actors and stakeholders though multiagency group panels 5. Improvement of facility infrastructure 6. Mentoring of junior health care workers.	There is need for research to: (1) Understand culturally appropriate interventions that can promote respectful maternity care (2) To examine the experience of pregnant women with disabilities /or/with children living with disabilities to improve our understanding on the scope of their blight. (3) To explore Facility preparedness of handling pregnant women living with disabilities. (4) To explore factors associated with disrespectful maternity care from the service provider’s point of view. (5) To establish prevalence of disrespectful care at the Facility level (6) To find out what tools are in place with healthcare providers

There is a need for a systemic and institutionalized effort beginning with policies and perceptions to address community-based socio-cultural norms such as the perception toward single and adolescent mothers. Emphasis should be put on both facility-based and individual-level health care educational training programs such as person-centred care to document professional association’s standards of care, and to implement and enforce these standards of care in all Maternity care facilities. For example, to combat the systemic negative attitudes and behaviors across some healthcare workers toward age and disability, curriculum and on-going training through coaching and mentorship could be implemented and directed toward cultural sensitivity and attitudes towards various groups. Reeducating healthcare workers at all levels about existing standards of care and instituting record-keeping to ensure the consistent adherence, monitoring, and enforcement of these standards would ensure that pregnant women and newborns are treated with dignity and respect while receiving obstetrical and neonatal care. To facilitate this, maternity healthcare staff must also be supported by the larger health care system and community as to the implementation and delivery of these standards through on-going training to ensure the appropriate delivery of care.

Maternity care facilities also require a system to monitor the delivery of care to ensure that patients are treated with kindness, respect, consideration, and professionalism. To reach that goal, staff attitudes towards patients and the way patients are treated needs to be a key element in both hiring and retention, and in the most egregious cases, abusers need to be reported to the police for redress under the law. A structure for reporting and response must be devised and instituted to ensure that when dealing with patients, all staff understand and carry out the principles of Respectful Care and gender responsiveness. As well, the fact that female health care workers were reported as being more likely than male healthcare workers to mistreat and abuse women under their care requires further study to determine the underlying factors for these attitudes and behaviours to better address this issue. These findings raise issues as to whether sufficient training and ongoing professional development for health care workers in Kenya is delivered, particularly around respectful maternity care and gender responsive services. This suggests the need to review the current curriculum both during training and for ongoing professional development to identify potential deficits and areas for improvement.

Finally, while addressing the larger structural barriers due to transportation and access are beyond this study, the structural deficits at facilities have been identified as a source of concern for both health care workers and patients. There are cases where there were not enough medications or even water for patients receiving maternity services. These barriers create increasingly difficult circumstances for healthcare workers to do their jobs in delivering respectful maternity care when they are also dealing with the lack of resources and supplies needed to competently provide necessary services. This is one of the implications of the Kenyan Free Maternity Care initiative that is creating greater demand for facility-based maternal services while the structural and material resources at facilities to deal with this increased demand has lagged. Addressing this structural barrier would assist healthcare workers in providing respectful care and serve to further reduce the maternal and child mortality rates [26].

### Study limitations

This study reported views from focus group discussions and interviews from a broad range of people with respect to maternal health care service delivery. This multifaceted approach strengthened the data quality and trustworthiness through the convergence of reports by these different groups and can be used to inform future work and potential areas for intervention in the Kenyan context. These findings are not intended to be generalizable in terms of statistical significance, but provide insight into the challenges with promoting facility prenatal, delivery and antenatal care in these two counties as well as across Kenya more generally.

Although data were collected from two research sites (Kisii and Kilifi), more data on the experience of disrespectful maternity care was reported by the participants from the Kisii context. While reports of disrespectful treatment were similar across both sites, women in Kilifi, a historically and culturally more Muslim community, were less forthcoming with providing stories detailing their experiences. In addition, religious leaders in both Kisii and Kilifi did not provide reports of disrespectful maternity care. Finally, in selecting participants for focus groups, male adolescents were not included which may limit the gender inclusivity of the overall study. We acknowledge that to have a more complete understanding on this topic, future work will also need the views of male adolescents and health care workers in these contexts regarding disrespectful maternity care.

## Conclusions

This paper presents data on women’s experiences of disrespectful care during pregnancy, labour and delivery. Findings established that the health care service industry’s culture of disrespect and abuse creates a hostile environment for women utilizing maternity health care facilities that discourage their future use for prenatal care and childbirth and the use by others. This not only jeopardizes improvements for maternal and neonatal outcomes but it also presents a significant barrier to the utilization of facility-based pregnancy and delivery services throughout Kenya. A concerted effort from relevant stakeholders is needed to develop policies, standards, and intervention tools that can ensure gender responsive and respectful care for all women during pregnancy, labour and delivery. Moreover, stakeholders across various organizations including Ministry of Health (MOH), Kenya Medical Association (KMA), National Nurses Association of Kenya (NNAK), and board members in individual healthcare facilities have a vested interest and must take coordinated action to rectify disrespectful attitudes and practices that currently permeate Kenya’s public maternal health care services. Innovative approaches to address this should take a systems approach to include routine training of health care workers for skills and compassionate care, and provision of incentives for those delivering at the healthcare facilities, which should be developed to integrate respectful maternity care as a routine quality component along a woman’s journey of pregnancy and delivery. In addition, addressing the broader work environment and structural barriers such as lack of basic equipment, inadequately staffed maternity wards, and limitations in beds and space which can contribute to a climate of disrespect and predisposes health care workers to moral distress will also serve to improve care and health outcomes.

## Supporting information

S1 Appendix Kilifi (Kaloleni) site–study tools and consent document (English, Swahili and Giriama). (DOCX)

S2 Appendix Kisii (Bomachoge Borabu) site–study and consent documents (English & Gusii). (DOCX)

S3 Appendix Barriers to skilled delivery service in Bomachoge-Borabu Kisii. (DOCX)

S4 Appendix Barriers to skilled delivery service in Kaloleni Kilifi. (DOCX)
