# Supplementary material for: Deliver on Your Own: Disrespectful Maternity Care in rural Kenya
Source: PLoS One. 2020 Jan 7;15(1):e0214836. doi: 10.1371/journal.pone.0214836 (PMC6946164; doi:10.1371/journal.pone.0214836)
Supplement: S1 Appendix — (DOCX) [file pone.0214836.s001.docx]

**Appendix 1: Kilifi (Kaloleni) site – Study Tools and consent document (English, Swahili and Giriama)**

**FGD Guides in English, Swahili and Giriama - Community Health Committee Participants**

**AQCESS Kenya Gender Assessment: FGD Guide for Community Health Committee Participants/Tathmini ya jinsia ya AQCESS Kenya: Muongozo wa FGD ya kamati ya afya ya anajamii manaoshiriki /Tathmini ya jinsia ya AQCESS Kenya: Muongozo wa FGD wa kamati ya afya ya wanajamii wanaoshiriki**

**Note to Facilitator:**Ensure participants meet the requirements in the recruitment form

- Follow the instructions indicated for each question
- Select a location that ensures privacy and space for participants to speak freely
- Welcome each participant as they arrive

**Introduction:**

- Start the FGD by reading the informed consent statement outlining all aspects of the study
- Present the assessment and objectives of the FGD to all participants
- Obtain informed consent from participants, including consent for audio recording (if consent is not provided, do not audio record)
- Prior or after the FGD, ask participants to fill in the participant information form (provide assistance as required). During this time, snacks/beverages can be distributed
- Introduce the facilitator and note-taker and ask participants to introduce themselves
- Share information with participants about the use of the recorder and who will hear this information
- Conduct a short icebreaker to help participants feel comfortable

**Overall Note:** For all questions, be sure to probe respondents for consideration around differences in age, disability, number and age of children, ethnicity and religion, level of wealth and education. /kumbusho ra jumula: maswali gosi ,hakikisha udzauza zaidhi anayehojiwa ukilola tofauti za ki miaka, ulemavu ,kiasi na namba ya ahoho, makabila na dhini, kiwango cha uthajiri na mashomo. Kumbusho kwa jumla: maswali yote hakikisha umeuliza Zaidi anayehojiwa ukiona tofauti za ki umri, kiasi na nambari ya watoto, makabila na dini ,kiwango cha masomo

| **Reference** | **Key Questions maswali muhumu/maswali muhimu** |
| --- | --- |
| **A. ACCESS TO RESOURCES, upatikanadzi wa rasilimali upatikanaji wa rasilimali** | |
| Access to Services/ upatikanadzi wa huduma/upatikanaji wa huduma | 1. Tell me a little bit about what the CHC does?   *Nambire machache zho CHC manahenda? / Nambie machache vile CHC wanafanya?*  What is your role on this committee?  *Una jukumu rani kwenye ino kamati? Una jukumu gani kwenye kamati hiyo?*   1. What MNCH services are available to women in your community?   *Ni huduma zani za MNCH ambazo zinapatikana kwa ana ache a kijiji kino? Ni huduma gani za MNCH ambazo zinapatikana kwa wanwake wa kijiji hiki?*   1. What types of services do they normally use?   *Kwa kawaidha ni aina yani ya huduma zinahumika? / Kwa kawaida ni aina gani ya huduma zinatumika?*   1. Are there services needed that are not available?   *Kuna huduma zozosi zinahitajika ambazo kaziho? Kala ndozho, ni hizo na mbona unaona kaziho? Kuna huduma zozote zinahitajika ambazo hazipo?*  If yes, what are they and why do you think they are not available? Kama ni kweli, ni zipi na mbona unaona hazipo ?   1. Are services available to all women (adolescents, disabled women, rich/poor, etc… **Probe respondents for consideration around differences in age, disability, number and age of children, ethnicity and religion, level of wealth and education.?** Why/Why not?   *Huduma zinapatikana kwa atu ache osi (vijana,ache enye ulemavu,mathajiri/maskini )? Mbona/mbona kaziho ? /huduma zinapatikana kwa wanawake wote(vijana,wanawake wenye ulemavu,matajiri/maskini)? Mbona /mbona hazipo?* |
| Barriers to Access/kuzuizi cha huduma/vizuizi vya huduma | 1. What are some of the challenges that you think women have in accessing services while they are pregnant?   *Ni changamoto hizo unafikiria atu ache manakirira kupata huduma wakathi mana mimba? Ni changamoto zipi unafikiria wanawake wanapitia kupata huduma wakati wana uja uzito?*   1. Are there reasons women do not go to the health facility during pregnancy?   What about to deliver their child?  If yes, what are they?  *Kuna sababu kwanoni kamenzi kwenda kituo cha afya wakathi mana mimba? Na wakathi wa kuzhala? Kala ndozho, ni ani hani? Kuna sababu kwanini hawataki kwenda kituo cha afya wakati wanauja uzito? Na wakati wa kuzaa? Kama ni kwelim ni akina nani?*   1. What about receiving services from a CHW/CMW? Are there reasons women do not receive these services?   *Na zino huduma za kuhokera akongo kumbola kwa CHW/CMW? Kuna sababu atu ache kamupata zizi huduma?/ na hizi huduma za kupokea wagonjwa kutoka CHW/CMW ? kuna sababu wanawake hamupati huduma hizi?*   1. If women do receive services from a CHW/CMW, are there challenges in benefitting from the services?   If yes, what are these challenges?  *Kala atu ache manapata zizi huduma kumbola CHW/CMW ,kuna changamoto za kunufaika kumbola kwa huduma zino kala nijeri, zo changamoto ni hizo? Kama wanawake wanapata huduma hizi kutoka kwa CHW/CMW , kuna changamoto za kunufaika kutoka kwa huduma hizi kama ni kweli,hizo changamoto ni zipi?*   1. Are there some women in the community that might have a different experience, either positive or negative? What barriers might they face in accessing services in a health facility?   What would you suggest to make it easier? (probe around age, ethnic group, disability, etc… **Probe respondents for consideration around differences in age, disability, number and age of children, ethnicity and religion, level of wealth and education.)**  *Kuna atu ache kijiji kino ambao makirira mautu tofauti ,either gaku banda moyo ama gakujenga moyo? Ni changamoto hizo manakirira kwa kupata huduma za kituo cha afya? Unga henza fu hende noni ili kurahisisha? / kuna wanawake kijiji hiki ambao wanapitia masuala tofauti ,aidha ya kuvunja moyo ama ya kujenga moyo? Ni changamoto zipi wanapitia kwa kupata huduma za kituo cha afya? Ungependa tufanye nini ili kurahisisha?* |
| Men’s Roles /jukumu ra alume/jukumu la wanaume | 1. Generally, how are men involved during ANC, Delivery or PNC?   *Kwa jumula, alume manadzi husisha dze wakathi wa ANC, kuzhala ama PNC? Kwa jumla, wanaume wanajihusisha vipi wakati wa ANC, kuzaa ama PNC ?*   1. Do men ever accompany women to a health facility for ANC   For delivery?  For PNC?  If not, why not?  *Alume ukala manahirika atu ache akwao kwa kituo cha afya ili ahenderwe ANC/kuzhala/PNC? Kala kamahenda mbona kamahenda? / wanaume huwa wanawapeleka wake wao kwa kituo cha afya ili wafanyiwe ANC/kuzaa/PNC?*   1. How do you think men could be supported to take a more active role in women’s pregnancy, delivery neo-natal and postnatal care?   *Unafikiria alume masaidhiwe hizho ili makale na bidhii katika jukumu ra ache Mario na mimba,kuzhala kwa neo natal na post natal care / unafikiria wanaume wasaidiwe vipi ili wawe na bidii katika jukumu la wanawake walio na uja uzito,kuzaa kwa neo natal na post natal care* |
| **B. Decision Making/kuhenda uamuzi kufanya uamuzi** | |
| Decision Making /kuhenda uamuzu /kufanaya uamuzi Processes and CHC Role jukumu na ngira za CHC /jukumu na njia za CHC | 1. Who generally makes the decision on whether or not to go for ANC in a typical household? What about to deliver or not deliver in a health facility?   *Kawaidha ho mudzini,ni hani ukala anahenda uamuzi kala undakwenda ama kundakwenda kwa ANC /kawaida hapo nyumbani ,ni nani huwa anafnya uamuzi kama utakwenda ama hutaenda kwa ANC?*   1. How does the CHC make decisions?   *CHC manahenda uamuzi kwa ngira yani? CHC wanafanya uamuzi kwa njia gani?*  How does the CHC address issues about accessibility so that all women can access services at a facility? With CHWs? *CHC ukala manaharira mautu ga upatadzi huduma, ili atu ache osi madime kupata huduma kwenye kituo cha afya CHC huwa wanachukulia vipi masuala ya upataji wa huduma ,ili wanawake waweze kupata huduma kwenye kituo cha afya ? je CHW nao?* |
|  | |
| Beliefs and Social Norms / Imani na masuala ga kijamii ga kawaidha/ masuala ya kijamii ya kawaida | Now, I would like to explore some of the beliefs that people have around marriage, family and breastfeeding.  *Vikara ninga henza kuelewa/ kumanya cho mnaamini kuhusu atu mario kwa ndoa,familia na kuamwa kwa ahoho./ sasa ningependa kuelewa/kujua mnaamini nini kuhusu watu walio kwa ndoa, familia na kunyonyesha watoto*   1. At what age do most men and women get married in this community? **(Probe around laws)**   *Ni umri wa miaka mingahi ukala alume na ache manahalana kijiji kino?( uza zaidhi na sheria za cho kijiji) / ni umri wa miaka mingapi huwa wanaume na wanawake wanaoana kijiji hiki?* (**uliza zaidi na sharia)**   1. What happens if someone outside of the age groups gets married (older or younger)? *Ukala kunahendeka noni kwa mutu amabaye kadzangwe kufikisha miaka ya kuhala/kuhalwa akihala/akihalwa(muthumia ama muhoho) huwa kunafanyika nini kwa mtu ambaye hajafikisha miaka ya kuoa/kuolewa akioa /akiolewa(mzee ama aliye mtoto)* 2. What about in terms of having children, what is the normal custom around the number of children and spacing of children? Who decides this generally? Na kuhusu masuala ga kuzhala ,kawaidha ni desturi ya ahoho angahi na muda wani baada ya kuzhala ndo andadima kugwira mimba kiheri/ na kuhusu masuala ya kuzaa,kawaida ni desturi ya watoto wangapi na muda upi baada ya kuzaa ndio anaweza kushika mimba tena? 3. What about if families do not want to have more children? What are the beliefs around use of contraception? (probe around community leaders messaging around this) na kala familia kazenzi kupata ahoho kiheri? Mnaamini noni kuhusu dawa za kupanga uzhazi(uza zaidhi kahi ya viongozi a kijiji manao sambaza habari zizi) / na kama familia hazitaki kupata watoto tena? Mnaamini nini kuhusu dawa za kupanga uzazi( uliza Zaidi kati ya viongozi wa kijiji wanao sambaza habari hizi) 4. Now, I would like to ask about circumcision, or cutting of females. What is the practice in this community? What are the reasons why it is practiced? Vikara ,ningahenza kuauza kuhusu kuthahiriwa ama kukeketa asichana. Mnahenda hizho kijiji kino? Sasa ningependa kuuliza kuhusu kupasha tohara ama kukuketa kwa wasichana. Mnafanya nini kijij hiki? 5. If a woman is circumcised (cut), does this affect where she gives birth? Kala mutu muche adzakekethrwa(kutoswa) ina athiri wakathi anazhala? Kama mwanamke amekeketwa (kukatwa) ina athiri wakati anazaa? 6. Do you think this or other practices may cause women harm? What are they? Why or why not? Unafikiria zoezi rino ama mazoezi mangine ganadima kuthuru atu ache? Ni mazoezi higo? Mbona na mbona kamuhenda? /unafikiria zoezi hili ama mazoezi mengine yanaweza kudhuru wanawake? Ni mazoezi yapi? 7. What is said in the community about the care a woman should receive during pregnancy, delivery and post-partum recovery?   *Kijiji kino mnaamba noni kuhusu huduma za muche wakathi wa mimba,wa kuzhala, na baada ya kuzhala? ?Kijiji hiki mnasema nini kuhusu huduma za mwanamke wakati ana uja uzito , wakati wa kuzaa na baada ya kuzaa*   1. What are babies (0-6 months) normally fed in this community? Is there a reason for this? If yes, what is the reason?   *Kawaidha Ahoho a miezi(0-6) manariswa noni kijiji kino? Kuna sababu yoyosi kuhusu zho? Kala nijeri ni kwanoni / kawaida watoto wa miezi (0-6) wanalishwa nini kijiji hiki? Kuna sababu yoyote? Kama ni kweli ni kwanini?* |
| **C. Practices and Participation ushiriki na mazoezi /ushiriki na mazoezi** | |
| CHC, Leadership and Governance CHC uongozi na uthawala / uongozi na utawala | I’d like to ask you a few questions about the roles and responsibilities of health service providers and health committees  *Ningahenza kukuza maswali machache kuhusu majukumu na wajibu wa manao lazha huduma za afya na kamati za afya / ningependa kukuuliza maswali machache kuhusu majukumu na wajibu wa wanaopeana huduma za afya na kamati za afya*   1. What do community leaders say about women’s participation in government and community structures?   Viongozi a kijiji kino manaamba noni kuhusu ushiriki wa atu ache kwa serikali na ujenzi/maenderero ga kijiji kino /viongozi wa kijiji hiki wanasema nini kuhusu ushiriki wa wanawake kwa serikalai na ujenzi/maendeleo ya kijiji hiki?   1. What about on this committee, how do you think you are perceived by the community?   *Unafikiria kamati inakumanya kwa ngira yani kijiji kino/ unafikiria kamati inakujua kwa njia gani kijiji hiki*   1. How do women and men become members of this committee? What kind of experience do they require?   *Unafikiria atu ache na alume manapatadze nafasi kutsagulwa anachama a kamati? Manamala atu Mario na kiwango/uzoefu wani / unafikiria wanawake na wanaume wanapaje nafasi kuchaguliwa wanachama wa kamati? Wanataka watu walio na kiwango/uzoefu gani?*   1. How do you think women’s participation in community health structures could be improved?   Unafikiria ushiriki wa atu ache kwenye ujenzi wa afya wa kijiji unadima kuboreshwa unafikiria ushiriki wa wanawake kwenye ujenzi wa afya wa kijiji unaweza kuboreshwa |
| **D. Policies, Rules and Program Messaging sera ,sheria na mapango wa kusambaza habari /sheria na mipango wa kusambaza habari** | |
| Health Messaging /habari za afya habari za afya | I would like to ask you about some of the messages you have heard through health promotion activities in your area.  *Ningahenza kukuza kuhusu habari udzizo sikira kukirira kwa kukuza kwa shughuli za afya manyumbani kwenu / hatimaye ningependa kukuuliza habari ulizoskia kupitia kwa kukuza kwa shughuli za afya manyumbani kwenu*   1. Have you heard health related messages on the radio, TV or in the community? Udzangwe kusikira habari zinazohusiana na afya kwa radio Tv ama kijijini thu? /ushawahi kusikia habari zinazohusiana na afya kwa radio,tv am kijijini tu? 2. What kind of messages did you hear?   *Wasikira habari hizo? Uliskia habari gani?*   1. Who were these messages targeting? Could you relate to the messages?   *Habari zino zinalenga anihani? Unadima kuelewa zo habari / habari hizo zinalenga akina nani? Unaweza kuelewa habari hizo?*   1. Where did you hear these messages? *(probe for television, radio, posters/print or community) wasikira hiko? (uza zaidhi kwa tv,radio,kwa mabango,ama kijijini) uliskia wapi? (uliza Zaidi kwa redio,tv amakwa mabango ama kijijini)* |
| Rules and Policies/ sheria na sera sheria na sera | 1. What are some of the rules or policies around MNCH services at health facilities?   *Ni sheria ama sera zani za huduma za MNCH kwa kituo cha afya/ ni sheria ama sera zipi zahuduma za MNCH kwa kituo cha afya?*   1. Do these make it easier or more difficult to access or use MNCH services? Explain.   *Zo sheria/sera zinarahisisha ama zinabuja kupata huduma ama kuhumira huduma za MNCH / hizo sheria /sera zinarahisisha ama kuleta vizuizi katika kupata huduma ama kutumia huduma za MNCH* |

**Closing: KUFUNGA**

1. Thank all participants
2. Remind participants of the information letter and how they can find out more or ask any questions
3. Gather all notes and materials

**FGD Guides in English, Swahili and Giriama -** **Male Participants**

**AQCESS Kenya Gender Assessment: FGD Guide for male Participants/tathmini ya jinsia ya AQCESS Kenya: Muongozo wa FGD wa alume manaoshiriki /tathmini ya jinsia ya AQCESS Kenya : muongozo wa wanaume wanaoshiriki**

- **Note to Facilitator:**Ensure participants meet the requirements in the recruitment form
- Follow the instructions indicated for each question
- Select a location that ensures privacy and space for participants to speak freely
- Welcome each participant as they arrive

**Introduction:**

- Start the FGD by reading the informed consent statement outlining all aspects of the study
- Present the assessment and objectives of the FGD to all participants
- Obtain informed consent from participants, including consent for audio recording (if consent is not provided, do not audio record)
- Prior or after the FGD, ask participants to fill in the participant information form (provide assistance as required). During this time, snacks/beverages can be distributed
- Introduce the facilitator and note-taker and ask participants to introduce themselves
- Share information with participants about the use of the recorder and who will hear this information
- Conduct a short icebreaker to help participants feel comfortable

**Overall Note:** For all questions, be sure to probe respondents for consideration around differences in age, disability, number and age of children, ethnicity and religion, level of wealth and education /kumbusho ra jumula: maswali gosi, hakikisha udzauza zaidhi anayehojiwa ukilola tofauti za ki miaka, ulemavu, kiasi na namba ya ahoho, makabila na dhini, kiwango cha uthajiri na mashomo. Kumbusho kwa jumla: maswali yote hakikisha umeuliza Zaidi anayehojiwa ukiona tofauti za ki umri, kiasi na nambari ya watoto, makabila na dini, kiwango cha masomo

| **Reference** | **Key Questions maswali muhimu /maswali muhimu** |
| --- | --- |
| **A. ACCESS TO RESOURCES upatikanadzi wa rasilimali/ upatikanaji wa rasilimali** | |
| Access to Services upatanadzi wa huduma /upatikanaji wa huduma | I would like to ask you about people’s experiences accessing and using health services in your community  *Ningahenza kuauza kuhusu mnago kirira ili kupata huduma na kuhumira huduma za afya kijiji henu / ningependa kuwauliza kuhusu mnayo yapitia ili kupata huduma na kutumia huduma za afya kijijini penu*   1. Can you describe people’s recent experience in going to a health facility in or near this community?   *Unadima kueleza mautu ga dzuzi dzuzi atu majamii ii marigokirira/marigoona marihokwenda sipitali kupata matibabu? Unaweza kunielezea mambo ya hivi karibuni ambayo watu wa kijiji hiki walipitia/waliyioyaona walipoenda kupata huduma kwenye kituo cha afya?*   1. What services are available there?   *Ni huduma hizo zinapatikana ko sipitali? Ni huduma gani zinapatikana huko hospitalini?*  What services are not available?  *Na ni huduma hizo zirizo kazipatikana? Na ni huduma zipi ambazo hazipatikani?*  What about accessing services from community health workers?  *Dze munapata huduma za afya kumbola kwa ahudumu a afya ya vidzidzini ? Je, munapata huduma za afya kutoka kwa wahudumu wa afya nyanjani/vijijini?*  What kind of services do you receive from them?  *Ni huduma hizo zilizo munapata kumbola kwa ahudumu a afya nyanjani/vijijini? Na ni huduma zipi ambazo munapata kutoka kwa wahudumu wa afya nyanjani/vijijini?*  How often?  *Huduma munazipata baada ya muda wani? Huduma hizo mwazipata kwa muda gani?*   1. What about health care for your wives or female family members, what kind of services are available for them?   *Ni huduma hizo za kiafya ache enu hedu anaache angine a familia zenu manapata? Ni huduma za afya bibi zenu ama wanawake wengine katika familia zenu wanapata?* |
| Barriers to Access vizuizi zha huduma/ vizuizi vya huduma  Practices and Participation mazoezi na ushiriki /mazoezi na ushiriki | 1. What are some of the challenges women (or female family member) face in getting checkups during pregnancy?   *Ni matatizo higo garigo anache manakirira/ manapata machenda sipitali kulolwa wakathi ana mimba? Ni changamoto zipi ambazo wanawake hupata wanapo enda kupata huduma za afya wakati wa uja uzito?*  What would you suggest to make it easier? (probe for challenges at the facility and from community)  *Kwa maoni igo, ungehenza kuhendedzwe kurahisisha anomane kupata huduma za afya wakati wa mimba? Kwa maoni yako, kungefanywa nini ili kurahisisha wamama kupata huduma za afya wakati wa uja uzito?*   1. Do women in this community deliver at a health facility? Can you tell me about this experience?   *Dze, anomaame kunokwenu manazharira sipitali? Unadima kunambira mambo garigo manakirira/managaona ko sipitali? Je wamama katika jamii hii hujifungua kwa kituo cha afya? Unaweza kutuelezea ni mambo gani wanayopitia wanapoenda kujifungua?*   - - Fees: Are the services free? How do prople pay for them? *Mariho: Huduma ni bule? Atu manarihiradze zo huduma? Malipo : Huduma ni bure? Wanalipiaje huduma hizo?*   - What other costs do people incur? Do people have to bring materials to the health facility? *Atu manarihishwa noni kaheri? Atu nikulagizwa mabebe vifaa zhozhosi kwa kituo cha afya / Watu hulipishwa nini tena/zaidi? Je watu huagizwa kuleta au kubeba vifaa vyovyote kwa kituo cha afya?*   - Distance: How do people reach the facility? How long did it take you? *Atu mafikadze kwa kituo cha afya? Manahumira muda wani kufika kwa kituo cha afya/ Watu hifika vipi kwa kituo cha afya? Watu hutumia muda gani kufika kituo cha afya?*   - Staff: How do the staff treat women? Do they receive services from a male or female provider? How does that make them feel? *Ahudumu aa afya anahudumia hizho anache? Anaache manahudumiwa ni ahudumu akiche hedu akilume? Akina mama huhudumiwa na wahudumu wa kiume ama wa kike? Na hiyo wanaichuliaje?*  1. Are women willing go to the facility again for delivery of another child? Why or why not? **(Probe around staff, age, ethnic group, distance**)   *Undamuja kiheri mucheo ko kituo cha afya ukazhale kiheri? Mbona ama mbona kundamuja?(uza zaidhi kuhusu ahendekazi, miaka, kabila, ure kwenda kituo cha afya) / Je, wamama wangependa kurudi tena kuzaa katika kituo cha afya? Mbona ama mbona hutamrudisha?* ***(uliza Zaidi kuhusu wafanyikazi,miaka,kabilam umbali wa kituo cha afya)***   1. Are there some families in the community that might have a different experience, either positive or negative? What barriers might they face in accessing services in a health facility? What would you suggest to make it easier? **(probe around age, ethnic group, disability, etc.)** .)   *Kuna famili zozosi kijiji kino ambao makirira vitu tofauti,either zha kubanda moyo ama kujenga moyo? Ni vizuizi zhani mnakirira ili kupata huduma za afya kwa kituo cha afya? Unaonerera noni kikihendwa kindarahisisha mautu?( Uza zaidhi kuhusu miaka,kabila,uremavu) / kuna familia zozote kijiji hiki ambao wanapitia vitu tofauti aidha vya kuvunja moyo ama kujenga moyo? Ni vizuizi vipi wanapitia ili kupata huduma za afya kwa kituo cha afya? Unaonelea nini kikifanywa kitarahisisha masuala haya? (uliza Zaidi kuhusu miaka,kabila ulemavu)*   1. For those whose deliver at home, what are some of the reasons why they did not go to a clinic? What prevents them from doing so?   *Kwa aryahu Mario manazharira midzini, ni sababu zani zirizo zinamahenda musende kuzharira sipitali? Ni noni ambacho kina mahenda masizharire sipitali? Kwa wale wanaozalia nyumbani ni sababu gani zinazowafanya wasiende kuzalia kwenye kituo cha afya ? ni nini ambacho kinawazuia?* |
| **B. Decision Making kuhenda uamuzi /kufanya uamuzi** | |
| Decision Making Processes ngira za kuhenda uamuzi / njia ya kufanya uamuzi | 1. In your families, who makes the decision on whether women should go or not to go for checkups during pregnancy? What about to deliver or not deliver in a health facility? Why?   *Kwa familia zenu ,ni nani hufanya uamuzi kama mama ataenda kliniki ama hataenda? Je swala la kuzaa nyumbani ama kuzaa kwa kituo cha afya? Kwanini? Kuno midzini ni hani ariye ni kuamuru kikala ano mame mandakwenda kiliniki hedu kamandakwenda? dze mambo ga kuzharira mudzini hedu sipitali kuno gakidze? Kwa noni?* |
| Male Support and Accompaniment usaidizi wa alume na kuongozana /usaidizi wa wanaume na kuongozana | 1. During the pregnancy, how are men involved? (probe around ANC, Delivery, PNC)   *Wakati wa mimba, kuzhala, na baada ya kuzhala, alume manahusika kihizho? Wakati wa uja uzito wako, wanaume wanahusika vipi? (uliza Zaidi kuhusu ANC, wakati wa kuzaa, PNC)*   1. Who accompanies women to the health facility during pregnancy or to deliver? Why this person?   *Nani kumhirika mama wa mimba klinik hedu sipitali wakati wa kudzala? Ni nani huambatana na mama mjamzito kwenda kwa kituo cha afya wakati wa uja uzito wake ama wakati wa kuzaa? Mbona mtu huyo?*   1. What other support do men offer during pregnancy? After pregnancy? Why? (probe around breastfeeding)   *Ni msaada hiyo alume nikulazha kwa anache wakati wa mimba na baada ya kuzhala? Kwanoni? Ni usaidizi gani mwengine ambao wanaume hupeana kwa mama wakati wa uja uzito wake? Baada ya uja uzito? Kwanini ? (uliza Zaidi kuhusu kunyonyesha )*   1. Do you think men can take a more active role in women’s pregnancy or neo-natal care? Why or Why not? *Unafikiria analume manadima kulazha usaidizi wakati wa mimba na wakati wakurera mwana wa tsini ya mwezi mwenga? Kwanoni? Kwanoni masidime?*   *Unafikiria wanaume wanaweza kujihusisha zaidi wanawake wakiwa na uja uzito ama wakati wa kumtunza motto mchanga chini ya mwezi mmoja? Kwanini? Kwanini wasiweze?* |
| **C. Beliefs and Perceptions Imani na mitazamo/ Imani na mitazamo** | |
| Beliefs and Social Norms Imani na mitazamo ya kijamii / Imani na mitazamo ya kijamii | 1. At what age do most men and women get married in this community?   *Ni umri wa miaka mingahi ukala alume na ache manahalana kijiji kino? Ni katika umri wa miaka mingapi huwa wanaume na wanawake wanaoana kijijini huku*?   1. What happens if someone outside of this age gets married (older or younger)?   *Dze, kundahendekanoni kikala mutu ambaye kadzangwe kufikisha miaka ya kuhala/kuhalwa akihala/akihalwa (muthumia ama muhoho)?*  Kutafanyika nini kama mtu aliye nje ya umri huu akioa (awe ni mzee ama wa umri wa chini)?   1. What about in terms of having children, what is the normal custom around the number of children and spacing of children?   *Na kuhusu masuala ga kuzhala, kawaidha ni desturi ya ahoho angahi na muda wani baada ya kuzhala ndo anadima kugwira mimba nyingine? / Na kuhusu masuala ya kuzaa, kawaida ni desturi ya watoto wangapi na muda upi baada ya kuzaa ndio anaweza kushika mimba tena?*  Who decides this generally?  *Ni zhani mwenye uamuzi kwa ujumula? / Ni nani mwenye uamuzi kwa jumla kuhusu mambo haya?*   1. What about if families do not want to have more children?   *Na kikala famili kamenzi kupata ahoho angine? Na kama familia hawataki kupata watoto wengine?*  What are the beliefs around use of contraception? (Probe around community leaders messaging around this)  *Dze atu kuuno manahairadze gano mautu gakuhumira ngira za kupanga uzhazi? (uza zaidhi kahi ya viongozi a kijiji manao sambaza habari zizi) / Je, fikra za watu kuhusu dawa za kupanga uzazi ni zipi katika kijiji hiki? (uliza zaidi kuhusu viongozi wa kijiji wanao sambaza habari hizi)*   1. Do you think there are any practices that may cause women harm? What are they? Why and how?   *Kuna mambo garigo ganadima kuthuru atu ache? Ni mambo higo? Kwanoni na Kidze?/Je, unafikiria kuna mambo yoyote yanayosababisha madhara kwa akina mama? Ni mambo yapi? Kwanini na kivipi?* |
| **E. Policies, Rules and Program Messaging sera, sheria na mpango wa kusambaza habari /sheria na mpango wa kusambaza habari** | |
| Health Messaging habari za afya /habari za afya | I would like to ask you about some of the messages you have heard through health promotion activities in your area. *Ningehenza kukuza kuhusu mambo urigogasikira kukirira kwa mpango wa kuelimisha atu madzumbani kwenu?/ Ningependa kukuuliza kuhusu baadhi ya habari ulizozisikia kupitia shughuli za uhamasisho wa afya manyumbani kwenu*   1. Have you heard health related messages on the radio, TV or in the community?   *Dze, wasikira masumuriro ga afya kukirira radio TV hedu hohenu kijijini? /Je, ushawahi kusikia maelezo ya afya kupitia kwa radio, TV ama kijijini kwenu?*   1. What kind of messages did you hear?   *Wasikira habari hizo? Ulisikia habari gani?*  Where did you hear these messages? *(Probe the medium for each message reported - for television, radio, posters/print or community) Wasikira hiko? (uza zaidhi kwa tv,radio,kwa mabango,ama kijijini) uliskia wapi? (uliza Zaidi kwa redio,tv amakwa mabango ama kijijini)*   1. Who were these messages targeting? Could you relate to the messages?   *Habari zino zalenga anihani? Wadima kuelewa habari zizo / Habari hizo zililenga akina nani? Ulielewa habari hizo?* |
| Rules and Policies sheria na sera | 4. What are some of the rules you know about at the health facility?  *Ni sheria zipi ambazo unazijua zilizoko kwenye kituo cha afya? / Ni sheria hizo ambazo unazimanya kuhusu kituo cha afya?*  5. Do these make it easier or more difficult to access or use MNCH services? Explain.  *Zinarahisisha ama zinabuja atu kupata huduma hedu kuhumira huduma za MNCH? / Zinarahisisha ama ziinaleta kizuizi katika kupata huduma za afya ama kutumia huduma za MNCH?* |

**Closing: Kufunga/Kufunga**

- Ask the participants if they have anything else they would like to contribute in terms of the discussion held
- Thank all participants for their time
- Remind participants that the information will remain anonymous and confidential and that they can find out more or ask any questions by calling the number on the information letter
- Gather all notes and materials and ensure they are properly labeled
- Stop the recording and ensure security of the file for translation/transcription

**FGD Guides in English, Swahili and Giriama - Female Participants**

**AQCESS Kenya Gender Assessment: FGD Guide for female Participants/tathmini ya jinsia ya AQCESS Kenya: Muongozo wa FGD wa atu ache manaoshiriki /tathmini ya jinsia ya AQCESS Kenya: muongozo wa FGD wa wanawake wanaoshiriki**

**Note to Facilitator:**

- Ensure participants meet the requirements in the recruitment form
- Follow the instructions indicated for each question
- Select a location that ensures privacy and space for participants to speak freely
- Welcome each participant as they arrive

**Introduction:**

- Start the FGD by reading the informed consent statement outlining all aspects of the study
- Present the assessment and objectives of the FGD to all participants
- Obtain informed consent from participants, including consent for audio recording (if consent is not provided, do not audio record)
- Prior or after the FGD, ask participants to fill in the participant information form (provide assistance as required). During this time, snacks/beverages can be distributed
- Introduce the facilitator and note-taker and ask participants to introduce themselves
- Share information with participants about the use of the recorder and who will hear this information
- Conduct a short icebreaker to help participants feel comfortable

**Overall Note:** **For all questions, be sure to probe respondents for consideration around differences in age, disability, number and age of children, ethnicity and religion, level of wealth and education.** /kumbusho ra jumula: maswali gosi ,hakikisha udzauza zaidhi anayehojiwa ukilola tofauti za ki miaka, ulemavu ,kiasi na namba ya ahoho, makabila na dhini, kiwango cha uthajiri na mashomo. Kumbusho kwa jumla: maswali yote hakikisha umeuliza Zaidi anayehojiwa ukiona tofauti za ki umri, kiasi na nambari ya watoto, makabila na dini ,kiwango cha masomo

| **Reference** | **Key Questions maswali muhimu/maswali muhimu** |
| --- | --- |
| **A. ACESS TO RESOURCES uapatikanadzi wa rasilimali/ upatikanaji wa rasilimali** | |
| Access to Services /upatikanadzi wa huduma/ upatikanaji wa huduma | I would like to ask you about people’s experiences accessing and using health services related to MNCH care.  *Ningaheza kuauza kuhusu mango kirira ili kupata huduma na kuhumira huduma za afya zinazohusiana na MNCH. Ningependa kuwauliza kuhusu masuala munayopitia ili kupata huduma na kutumia huduma za afya zinazohusiana na MNCH.*  1. Can you describe people’s recent experience in going to a health facility in or near this community?  *Unadima kueleza mautu ga dzuzi dzuzi atu majamii ii marigokirira/marigoona marihokwenda sipitali kupata matibabu? Unaweza kunielezea mambo ya hivi karibuni ambayo watu wa kijiji hiki walipitia/waliyioyaona walipoenda kupata huduma kwenye kituo cha afya?*  2. What services are available there?  *Ni huduma hizo zinapatikana ko sipitali? Ni huduma gani zinapatikana huko hospitalini?*  What services are not available?  *Na ni huduma hizo zirizo kazipatikana? Na ni huduma zipi ambazo hazipatikani?*  What about accessing services from community health workers?  *Dze munapata huduma za afya kumbola kwa ahudumu a afya ya vidzidzini ? Je, munapata huduma za afya kutoka kwa wahudumu wa afya nyanjani/vijijini?*  What kind of services do you receive from them?  *Ni huduma hizo zilizo munapata kumbola kwa ahudumu a afya nyanjani/vijijini? Na ni huduma zipi ambazo munapata kutoka kwa wahudumu wa afya nyanjani/vijijini?*  How often?  *Huduma munazipata baada ya muda wani? Huduma hizo mwazipata kwa muda gani?* |
| Barriers to Access kizuizi cha huduma/ kizuizi cha huduma | 1. What are some of the challenges women (or female family member) face in getting checkups during pregnancy?   *Ni matatizo higo garigo anache manakirira/ manapata machenda sipitali kulolwa wakathi ana mimba? Ni changamoto zipi ambazo wanawake hupata wanapo enda kupata huduma za afya wakati wa uja uzito?*  What would you suggest to make it easier? (probe for challenges at the facility and from community)  *Kwa maoni igo, ungehenza kuhendedzwe kurahisisha anomane kupata huduma za afya wakati wa mimba? Kwa maoni yako, kungefanywa nini ili kurahisisha wamama kupata huduma za afya wakati wa uja uzito?*   1. Do women in this community deliver at a health facility? Can you tell me about this experience?   *Dze, anomaame kunokwenu manazharira sipitali? Unadima kunambira mambo garigo manakirira/managaona ko sipitali? Je wamama katika jamii hii hujifungua kwa kituo cha afya? Unaweza kutuelezea ni mambo gani wanayopitia wanapoenda kujifungua*?   - - Fees: Are the services free? How do prople pay for them? *Mariho: Huduma ni bule? Atu manarihiradze zo huduma? Malipo : Huduma ni bure? Wanalipiaje huduma hizo?*   - What other costs do people incur? Do people have to bring materials to the health facility? *Atu manarihishwa noni kaheri? Atu nikulagizwa mabebe vifaa zhozhosi kwa kituo cha afya / Watu hulipishwa nini tena/zaidi? Je watu huagizwa kuleta au kubeba vifaa vyovyote kwa kituo cha afya?*   - Distance: How do people reach the facility? How long did it take you? *Atu mafikadze kwa kituo cha afya? Manahumira muda wani kufika kwa kituo cha afya/ Watu hifika vipi kwa kituo cha afya? Watu hutumia muda gani kufika kituo cha afya?*   - Staff: How do the staff treat women? Do they receive services from a male or female provider? How does that make them feel? *Ahudumu aa afya anahudumia hizho anache? Anaache manahudumiwa ni ahudumu akiche hedu akilume?/ Akina mama huhudumiwa na wahudumu wa kiume ama wa kike? Na hiyo wanaichuliaje?*  1. Are women willing go to the facility again for delivery of another child? Why or why not? **(probe around staff, age, ethnic group, distance)**   *Undamuja kiheri mucheo ko kituo cha afya ukazhale kiheri? Mbona ama mbona kundamuja?(uza zaidhi kuhusu ahendekazi, miaka, kabila, ure kwenda kituo cha afya) / Je, wamama wangependa kurudi tena kuzaa katika kituo cha afya? Mbona ama mbona hutamrudisha? (uliza Zaidi kuhusu wafanyikazi, miaka,kabilam umbali wa kituo cha afya)*   1. Are there some families in the community that might have a different experience, either positive or negative? What barriers might they face in accessing services in a health facility? What would you suggest to make it easier? **(probe around age, ethnic group, disability, etc**.)   *Kuna famili zozosi kijiji kino ambao makirira vitu tofauti, either zha kubanda moyo ama kujenga moyo? Ni vizuizi zhani mnakirira ili kupata huduma za afya kwa kituo cha afya? Unaonerera noni kikihendwa kindarahisisha mautu?* ***(Uza zaidhi kuhusu miaka,kabila,uremavu)*** */ kuna familia zozote kijiji hiki ambao wanapitia vitu tofauti aidha vya kuvunja moyo ama kujenga moyo? Ni vizuizi vipi wanapitia ili kupata huduma za afya kwa kituo cha afya? Unaonelea nini kikifanywa kitarahisisha masuala haya?* ***(uliza Zaidi kuhusu miaka,kabila ulemavu)***   1. For those whose deliver at home, what are some of the reasons why they did not go to a clinic? What prevents them from doing so?   *Kwa aryahu Mario manazharira midzini, ni sababu zani zirizo zinamahenda musende kuzharira sipitali? Ni noni ambacho kina mahenda masizharire sipitali? Kwa wale wanaozalia nyumbani ni sababu gani zinazowafanya wasiende kuzalia kwenye kituo cha afya? Ni nini ambacho kinawazuia?*   1. After birth, who checks mothers? Where does this checkup take place? Is it by a health professional? *Baada ya kuzhala, ni zhani nikumpima mame mzhazi? Anapimirwa hiko? Dze, anapimwa ni mhudumu wa afya? / Baada ya kuzaa, ni nani humkagua mama? Ukaguzi hufanyiwa wapi? Je, ukaguzi hufanywa na mhudumu wa afya?* |
| **B. Decision Making/kuhenda uamuzi / kufanya uamuzi** | |
| Decision Making Processes ngira za kuhenda uamuzi / njia za kufanya uamuzi | 1. In your families, who makes the decision on whether women should go or not to go for checkups during pregnancy? What about to deliver or not deliver in a health facility? Why?   *Kwa familia zenu ,ni nani hufanya uamuzi kama mama ataenda kliniki ama hataenda? Je swala la kuzaa nyumbani ama kuzaa kwa kituo cha afya? Kwanini? Kuno midzini ni hani ariye ni kuamuru kikala ano mame mandakwenda kiliniki hedu kamandakwenda? dze mambo ga kuzharira mudzini hedu sipitali kuno gakidze? Kwa noni?*   1. In which areas do you feel women can influence decisions in their household? **(Probe for age of marriage, nutrition, breastfeeding, seeking health care, etc.)**   *Ni mambo higo ga kwako nyumbani ambago unadima kukala na ushawishi? (Uza zaidhi kwa miaka ya kuhala/kuhalwa,lishe bora,kuamwisa mhoho,kutafuta huduma za afya) / ni sehemu gani unafikiria wamama wanaweza kufanya ushawishi katika maamuzi kwao nyumbani?* ***(Uliza Zaidi kwa miaka ya kuoa/kuolewa, kunyonyesha mtoto ,kutafuta huduma za afya)***   1. What do you think would help in terms of increasing women’s ability to make more decisions about their own health?   *Unafikiria ninoni kindaasaidhia kwa masuala ga kungeza uwezo wako wa kuhenda uamuzi kuhusu afya ya kwako? Unafikiria ni nini kinaweza kuongeza uwezo wa kina mama wa kufanya uamuzi kuhusu afya zao?* |
| Male Support and Accompaniment usaidzizi wa alume nakuongozana / usaidizi wa wanaume nakuongozana | 1. During the pregnancy, how are men involved? (probe around ANC, Delivery, PNC)   *Wakati wa mimba, kuzhala, na baada ya kuzhala, alume manahusika kihizho? Wakati wa uja uzito wako, wanaume wanahusika vipi? (uliza Zaidi kuhusu ANC,wakati wa kuzaa,PNC)*   1. Who accompanies women to the health facility during pregnancy or to deliver? Why this person?   *Nani kumhirika mama wa mimba klinik hedu sipitali wakati wa kudzala? Ni nani huambatana na mama mjamzito kwenda kwa kituo cha afya wakati wa uja uzito wake ama wakati wa kuzaa? Mbona mtu huyo?*   1. What other support do men offer during pregnancy? After pregnancy? Why? (probe around breastfeeding) *Ni msaada hiyo alume nikulazha kwa anache wakati wa mimba na baada ya kuzhala? Kwanoni? Ni usaidizi gani mwengine ambao wanaume hupeana kwa mama wakati wa uja uzito wake? Baada ya uja uzito? Kwanini ? (uliza Zaidi kuhusu kunyonyesha )* 2. Do you think men can take a more active role in women’s pregnancy or neo-natal care? Why or Why not?   *Unafikiria analume manadima kulazha usaidizi wakati wa mimba na wakati wakurera mwana wa tsini ya mwezi mwenga? Kwanoni? Kwanoni masidime?*  *Unafikiria wanaume wanaweza kujihusisha zaidi wanawake wakiwa na uja uzito ama wakati wa kumtunza motto mchanga chini ya mwezi mmoja? Kwanini? Kwanini wasiweze?* |
| **C. Beliefs and Perceptions imani na mitazamo /Imani na mitazamo** | |
| Beliefs and Social Norms imani na masuala ga kijamii / Imani na masuala ya kijamii | Now, I would like to explore some of the beliefs that people have around marriage, family and breastfeeding. *Vikara ninga henza kuelewa/kumanya cho mnaamini kuhusu atu mario kwa ndoa, familia na kuamwa kwa ahoho/ Sasa ningependa kuelewa/kujua fikra ambazo watu wanazo kuhusu ndoa, familia na kunyonyesha watoto.*   1. At what age do most men and women get married in this community?   *Ni umri wa miaka mingahi ukala alume na ache manahalana kijiji kino? Ni katika umri wa miaka mingapi huwa wanaume na wanawake wanaoana kijijini huku?*   1. What happens if someone outside of this age gets married (older or younger)?   *Dze, kundahendekanoni kikala mutu ambaye kadzangwe kufikisha miaka ya kuhala/kuhalwa akihala/akihalwa (muthumia ama muhoho)?*  *Kutafanyika nini kama mtu aliye nje ya umri huu akioa (awe ni mzee ama wa umri wa chini)?*   1. What about in terms of having children, what is the normal custom around the number of children and spacing of children?   *Na kuhusu masuala ga kuzhala, kawaidha ni desturi ya ahoho angahi na muda wani baada ya kuzhala ndo anadima kugwira mimba nyingine? / Na kuhusu masuala ya kuzaa, kawaida ni desturi ya watoto wangapi na muda upi baada ya kuzaa ndio anaweza kushika mimba tena?*  Who decides this generally?  *Ni zhani mwenye uamuzi kwa ujumula? / Ni nani mwenye uamuzi kwa jumla kuhusu mambo haya?*   1. What about if families do not want to have more children?   *Na kikala famili kamenzi kupata ahoho angine? Na kama familia hawataki kupata watoto wengine?*  What are the beliefs around use of contraception? (probe around community leaders messaging around this)  *Dze atu kuuno manahairadze gano mautu gakuhumira ngira za kupanga uzhazi? (uza zaidhi kahi ya viongozi a kijiji manao sambaza habari zizi) / Je, fikra za watu kuhusu dawa za kupanga uzazi ni zipi katika kijiji hiki? (uliza zaidi kuhusu viongozi wa kijiji wanao sambaza habari hizi)*   1. Now, I would like to ask about circumcision, or cutting of females. What is the practice in this community? What are the reasons why it is practiced?   *Vikara, ningahenza kuauza kuhusu kuthahiriwa ama kukeketa asichana. Mnahenda hizho kijiji kino? Sasa ningependa kuuliza kuhusu kupasha tohara kwa wasichana. Huwa inafanyika vipi hapa kwenu?*  If a woman is circumcised (cut), does this affect where she gives birth?  *Kala mame watahiriwa, hino inadima kumhenda akale kuna kuthu ariko kadima kwenda zhala? Kama mwanamke amepashwa tohara, inaweza kumfanya ajifungulie mahali maalum tu*?   1. Do you think this or other practices may cause women harm? What are they? Why or why not?   *Unafikiria ino hedu mambo garigo ganadima kuthuru atu ache? Ni mambo higo? Kwanoni na Kidze?/Unafikiria hii ama kuna mambo mengine ambayo yanaweza kusababisha madhara kwa akina mama? Ni mambo yapi? Kwanini na kivipi?* |
| **D. Practices and Participation mazoezi na ushiriki / mazoezi na ushiriki** | |
| Breastfeeding Practices  + Norms on Breastfeeding zoezi ra kuamwisa + masuala ga kijamii ga kuamwisa/ zoezi la kunyonyesha + masuala ya kijamii ya kunyonyesha | We are going to talk about feeding of children under 6 months.  *Sasa tutaongea kuhusu jinzi ya kuwalisha watoto walio na umri wa chini ya miezi 6.*   1. What do women normally feed their children who are under 6 months? Why?   *Ano mame nikwapa ahoho mario chini ya miezi 6 chakurya hicho? Kwanoni? Kwa kawaida, akina mama huwalisha nini watoto wenye umri chini ya miezi 6? Kwanini?*   1. Where do women first learn about the practices you described?   *Anomame nikushomerahi gano mautu gakupa chakurya ahoho? / Wamama hujifunza/hujulia wapi kuhusu kumlisha mtoto kama ulivyo tuelezea?*  What are some of the challenges faced in breastfeeding children? Why do you think this is? **(Probe for time to breastfeed, support from male family members, physical challenges)**  Dze, ni changamoto hizo anomame nikupata makikalama mana a mwisa anaao? Unaona ni kwanoni? **(Uza zaidhi kwa muda wa kuamwisa,msaada kumbola kwa alume aa famili ,changamoto za kimwiri)** / changamoto zipi ambazo wamama wanapitia wakati wananyonyesha watoto wao? Munaona ni kwanini? **( uliza Zaidi kwa muda wa kunyonyesha, msaada kutoka kwa wanaume wa familia, changamoto za kimwili)** |
| **E. Policies, Rules and Program Messaging sera,sheria na mpango wa kusambaza habari/ sera,sheria na mpango wa kusambaza habari** | |
| Health Messaging habari za afya/ habari za afya | I would like to ask you about some of the messages you have heard through health promotion activities in your area. *Ningehenza kukuza kuhusu mambo urigogasikira kukirira kwa mpango wa kuelimisha atu madzumbani kwenu?/ Ningependa kukuuliza kuhusu baadhi ya habari ulizozisikia kupitia shughuli za uhamasisho wa afya manyumbani kwenu*   1. Have you heard health related messages on the radio, TV or in the community?   *Dze, wasikira masumuriro ga afya kukirira radio TV hedu hohenu kijijini? /Je, ushawahi kusikia maelezo ya afya kupitia kwa radio, TV ama kijijini kwenu?*   1. What kind of messages did you hear?   *Wasikira habari hizo? Ulisikia habari gani?*  Where did you hear these messages? (Probe the medium for each message reported - for television, radio, posters/print or community)  *Wasikira hiko? (uza zaidhi kwa tv,radio,kwa mabango,ama kijijini) uliskia wapi? (uliza Zaidi kwa redio,tv amakwa mabango ama kijijini)*  Who were these messages targeting? Could you relate to the messages?  *Habari zino zalenga anihani? Wadima kuelewa habari zizo / Habari hizo zililenga akina nani? Ulielewa habari hizo?* |
| Rules and Policies sheria na sera | 3. What are some of the rules you know about at the health facility?  *Ni sheria zipi ambazo unazijua zilizoko kwenye kituo cha afya? / Ni sheria hizo ambazo unazimanya*  *kuhusu kituo cha afya?*  Do these make it easier or more difficult to access or use MNCH services? Explain. *Zinarahisisha ama zinabuja atu kupata huduma hedu kuhumira huduma za MNCH? / Zinarahisisha ama ziinaleta kizuizi katika kupata huduma za afya ama kutumia huduma za MNCH?* |

**Closing: KUFUNGA /kufunga**

- Ask the participants if they have anything else they would like to contribute in terms of the discussion held
- Thank all participants for their time
- Remind participants that the information will remain anonymous and confidential and that they can find out more or ask any questions by calling the number on the information letter

**AQCESS Kenya Gender Assessment: KII with stakeholders**

Key informant interviews are semi-structured interviews aimed at getting the perspective of various issues from knowledgeable informants. A list of KIIs will be provided to the interviewer.

**Note to Facilitator:**

- Ensure focus is on building rapport with the interviewee
- Note down any probing questions that are asked

**Introduction**

- Introduce yourself and Present the assessment and objectives of the KII
- Read the information letter and consent form
- Obtain informed consent from respondent, including consent for audio recording (if consent is not provided, do not audio record)
- Ask if the respondent has any questions before beginning the interview

**KII – Religious Leader**

| **INTRODUCTION** | |
| --- | --- |
| Introduction | Thank you for meeting with me today. I would like to start with getting to know you a little better. Could you tell how long have you lived in this community?  How long you have been the religious leader in this community?  *Asanteni kwa kukutana na mimi leo, ningependa nikujue zaidi. Unaweza kuniambia muda umeishi kwa jamii hii, na muda gani umekua kiongozi wa dini kwenye jamii hii?*   1. What is the main religion practiced in this community?   *Ni dini ipi inafuatwa sana kwenye jamii hii?*   1. As a religious leader in this community, what is your role?   *Kama kiongozi wa dini katika jamii hii, jukumu lako ni lipi?* |
| **B. DECISION MAKING /kuweka maamuzi** | |
| How can women’s participation in decision-making at the household and community levels be improved? | 1. In your opinion, what is the main role of women and men in the household? *Kwa maoni yako ni jukumu gani kubwa la wanawake na wanaume katika nyumba?*   What about in the community?  *Na je kwenye jamii nayo?*   1. When it comes to decision making in the household, who should be making decisions generally?   *Ikifika katika kufanya maamuzi kwenye nyumba, ni nani wa kufanya maamuzi kwa kawaida?*  What about when it comes to issues around pregnancy and delivery?  *Na ikiwa kwa masuala ya uja uzito na uzazi ni nani hufanya maamuzi?*   1. Are there things that you recommend as a religious leader to either men or women about this?   *Je, kuna mambo wewe kama kiongozi wa dini unaweza kupendekeza kwa wanawake ama wanaume kuhusu kufanya maamuzi nyumbani?* |
| **C. BELIEFS AND PERCEPTIONS / Imani na vile uonavyo** | |
| What are the beliefs and social norms around family planning and its use? In relation to spacing of children? | 1. What are the beliefs around family planning in this community?   *Ni fikra gani kuhusu upangaji wa uzazi ziko kwenye jamii hii?*   1. As a religious leader, what messaging do you provide around family planning?   *Kama kiongozi wa dini, ni jumbe gani unazopeana kwa watu kuhusu upangaji wa uzazi katika jamii hii?*   1. What about the beliefs around when to have children and how often?   *Je kuhusu fikra za ni wakati gani wa kupata watoto na baada ya muda gani?* |
| What are the social beliefs about age of marriage, FGM, and how are these beliefs or norms enforced? | 1. Could you tell me a little bit about marriage practices in the community?   *Unaweza kunieleza kidogo kuhusu masuala ya ndoa kwenye jamii hii?*  At what age do people generally get married?  *Watu huwa wanaoana wakiwa na umri gani?*  Is it the same for men and women?  *Na je umri huo ni sawa kwa wanawake na wanaume?*  Why? *Kwanini?*   1. What happens if someone outside of this age gets married (older or younger)?   *Kutafanyika nini kama mtu aliye nje ya umri huu akioa (awe ni mzee ama wa umri wa chini)*   1. As a religious leader, what messaging do you provide around having and spacing children   *Kama kiongozi wa dini, ni maelezo gani unapeana kuhusu kupata watoto na upangaji wa uzazi?*   1. What are some of the main practices around female genital cutting in this community?   *Huwa mnafanya nini kuhusu masuala ya kupasha tohara kwa watoto wa kike kwenye jamii hii?*   1. In your opinion, what are the reasons for this?   *Kwa maoni yako unaona ni kwa nini?* |
| What is the perceived role of men in supporting access and use of MNCH services? (incl. accompaniment) | I would like to ask about men’s roles in this community when it comes to supporting access to health services for women during pregnancy and delivery  *Je, jukumu la wanaume katika jamii hii kwenye maswala ya kupokea huduma za afya wakati wa uja uzito na kujifungua kwa kina mama ni lipi?*   1. How do husbands or male family members generally support women in accessing ANC/Delivery/PNC?   *Je, mabwana ama waume wanasaidia vipi wanawake ili waweze kufikia huduma za afya wakati wakati wa uja uzito, kujifungua, na huduma za baada ya kujifungua?*   1. Are men encouraged to play this role?   *Je, wanaume wanahimiwa wafanye hivyo?*  If yes, how?  *Kama ni kweli, kivipi?*  If no, why not?  *Kama hawaambiwi, kwanini?*   1. What would prevent men from playing an active role in supporting women’s health issues?   *Kinacho zulia wanaume wasisaidie katika masuala ya afya ya wanawake ni nini?* |

**KII – Community Leader/Elder and Local Government Leader / Kiongozi wa jamii /kiongozi wa serikali**

| **INTRODUCTION /kutambulishwa** | |
| --- | --- |
| Introduction | Thank you for meeting with me today. I would like to start with getting to know you a little better. Could you tell how long have you lived in this community?  *Asante kwa kukutana na mimi leo, unaweza kunieleza ni muda gani umeishi kwa jamii hii?*  How long have you been a leader in this community?  *Na muda gani umekua kiongozi wa jamii hii?*   1. As a leader in this community, what is your role?   *Kama kiongozi katika jamii hii, jukumu lako ni lipi?* |
| **B. DECISION MAKING /kufanya uamuzi** | |
| How can women’s participation in decision-making at the household and community levels be improved? | 1. In your opinion, what is the main role of women and men in the household? *Kwa maoni yako ni jukumu gani kubwa la wanawake na wanaume katika nyumba?*   What about in the community?  *Na je kwenye jamii nayo?*   1. When it comes to decision making in the household, who should be making decisions generally?   *Ikifika katika kufanya maamuzi kwenye nyumba, ni nani wa kufanya maamuzi kwa kawaida?*  What about when it comes to issues around pregnancy and delivery?  *Na ikiwa kwa masuala ya uja uzito na uzazi ni nani hufanya maamuzi?*   1. Are there things that you recommend as a leader to either men or women about this?   *Je, kuna mambo wewe kama kiongozi unaweza kupendekeza kwa wanawake ama wanaume kuhusu kufanya maamuzi nyumbani?* |
| **C. BELIEFS AND PERCEPTIONS /Imani na unavyoona** | |
| What are the beliefs and social norms around family planning and its use? In relation to spacing of children? | 1. What are the beliefs around family planning in this community?   *Ni fikra gani kuhusu upangaji wa uzazi ziko kwenye jamii hii?*   1. As a leader, what messaging do you provide around family planning?   *Kama kiongozi, ni jumbe gani unazopeana kwa watu kuhusu upangaji wa uzazi katika jamii hii?*   1. What about the beliefs around when to have children and how often?   *Je kuhusu fikra za ni wakati gani wa kupata watoto na baada ya muda gani?* |
| What are the social beliefs about age of marriage, FGM, and how are these beliefs or norms enforced? | 1. Could you tell me a little bit about marriage practices in the community?   *Unaweza kunieleza kidogo kuhusu masuala ya ndoa kwenye jamii hii?*  At what age do people generally get married?  *Watu huwa wanaoana wakiwa na umri gani?*  Is it the same for men and women?  *Na je umri huo ni sawa kwa wanawake na wanaume?*  Why? *Kwanini?*   1. What happens if someone outside of this age gets married (older or younger)?   *Kutafanyika nini kama mtu aliye nje ya umri huu akioa (awe ni mzee ama wa umri wa chini)*   1. As a leader, what messaging do you provide around having and spacing children   *Kama kiongozi, ni maelezo gani unapeana kuhusu kupata watoto na upangaji wa uzazi?*   1. What are some of the main practices around female genital cutting in this community?   *Huwa mnafanya nini kuhusu masuala ya kupasha tohara kwa watoto wa kike kwenye jamii hii?*  In your opinion, what are the reasons for this?  *Kwa maoni yako unaona ni kwa nini?* |
| What is the perceived role of men in supporting access and use of MNCH services? (incl. accompaniment). | I would like to ask about men’s roles in this community when it comes to supporting access to health services for women during pregnancy and delivery  *Je, jukumu la wanaume katika jamii hii kwenye maswala ya kupokea huduma za afya wakati wa uja uzito na kujifungua kwa kina mama ni lipi?*   1. How do husbands or male family members generally support women in accessing ANC/Delivery/PNC?   *Je, mabwana ama waume wanasaidia vipi wanawake ili waweze kufikia huduma za afya wakati wakati wa uja uzito, kujifungua, na huduma za baada ya kujifungua?*   1. Are men encouraged to play this role?   *Je, wanaume wanahimiwa wafanye hivyo?*  If yes, how?  *Kama ni kweli, kivipi?*  If no, why not?  *Kama hawaambiwi, kwanini?*   1. What would prevent men from playing an active role in supporting women’s health issues?   *Kinacho zuilia wanaume wasisaidie katika masuala ya afya ya wanawake ni nini?* |

**KII – Health Facility Personnel**

| **INTRODUCTION** | |
| --- | --- |
| Introduction | Thank you for meeting with me today. I would like to start with getting to know you a little better. Could you tell me how long you have worked at this health facility and what is your role? |
| **A. ACCESS TO HEALTH RESOURCES** | |
| Availability of Services | 1. To start, can you tell me about some of the MNCH services that are available at this facility? 2. How well do the current health services available at this clinic respond to women’s and men’s needs? **Probe respondent for consideration around differences in age, disability, number and age of children, ethnicity and religion, level of wealth and education** 3. Are there services not provided that should be?   If yes, what are they? |
| Challenges/Barriers | 1. In your opinion, what are the challenges that women in this community face in accessing MNCH care at the facility level? 2. What barriers do you see for women in attending ANC services? Delivery in a facility?   *Probe respondents for consideration around differences in age, disability, number and age of children, ethnicity and religion, level of wealth and education*   1. How do you think these barriers can be addressed? |
| **C. BELIEFS AND PERCEPTIONS** | |
| Beliefs around MNCH Care | 1. Why do you think women do not deliver in health facilities? Are there any social reasons? 2. Are there differences between younger women (adolescents) and older women (or those that have multiple children)? **Probe respondent for consideration around differences in age, disability, number and age of children, ethnicity and religion, level of wealth and education**   What are these? |
| What are the beliefs and social norms around family planning and its use? In relation to spacing of children? | 1. What are the beliefs around family planning in this community? 2. As a health professional, what messaging do you provide around family planning at the clinic? |
| **D. PRACTICES/mazoezi** | |
| What is the practice of men in supporting access and use of MNCH services? (incl. accompaniment). | Now, I would like to ask about men’s roles in this community when it comes to supporting access to health services for women during pregnancy and delivery.   1. Do husbands or male family members generally accompany their wives/female family members to ANC visits?   For Delivery?  For PNC?  Why or Why not?   1. What are some of the rules of the facility around this? |
| Health Personnel Treatment | 1. In your experience, how do male (and then for female) clients treat you? 2. What is considered respectful treatment of male and or female clients? |
| Exclusive Breastfeeding | 1. What are the customs around breastfeeding in this community? 2. When a woman delivers in the facility, what is she instructed to do in terms of feeding her newborn? |
| **E. POLICIES, RULES** | |
| Rules/Policies | 1. Are there specific policies at the facility in regards to who can access services? Adolescents? Poor? Disabled? **Probe respondent for consideration around differences in age, disability, number and age of children, ethnicity and religion, level of wealth and education** 2. For staff and management, are there any policies related to safe working environments in this facility?   What are they?   1. What about personal leave?   How is this applied? |
| Health Facility Governance | 1. What are the tasks performed by male and female health providers?   Is there a difference between those performed by men and those by women?  ?   1. Tell me a little about the governance structures at these facilities?   Who sits on these committees?  What are their roles |

**KII – Community Health Worker**

| **INTRODUCTION** | |
| --- | --- |
| Introduction | Thank you for meeting with me today. I would like to start with getting to know you a little better. Could you tell me how long you have been a CHW?  *Asante kwa kukutana na mimi leo, unaweza kunieleza ni muda gani umehudumu kama CHW?*  What is your role?  *Kama kiongozi katika jamii hii, jukumu lako ni lipi?* |
| **A. ACCESS TO HEALTH RESOURCES** | |
| Availability of Services | 1. To start, can you tell me about what type of services a CHW provides to women and children?   *Tukianzia, unaweza kunieleza ni huduma gani unapeana kwa akina mama na watoto kama CHW?*   1. How well would you say that these services respond to women’s and children’s needs?   *Je, hizi huduma zinatosheleza mahitaji ya akina mama na watoto?*  **Probe respondents for consideration around differences in age, disability, number and age of children, ethnicity and religion, level of wealth and education**   1. Are there services not provided that should be?   *Kuna huduma ambazo ni muhimu na hazipatikani?*  If yes, what are they?  *Kama zipo, ni zipi?*  Are there needs not covered in this community?  *Kuna mahitaji ya afya ya akina mama na watoto ambayo hayajaangaziwa huduma?*  If yes, what are they?  *Kama zipo, ni zipi?* |
| Challenges/Barriers | 1. In your opinion, what are the challenges that women in your community face in accessing MNCH care in the community?   *Kwa maoni yako, ni changamoto zipi ambazo wanawake wanapitia ili kufikia huduma za MNCH kwenye jamii hii?*   1. What barriers do you see for women in attending ANC services?   *Ni vizuizi gani ambavyo vinazuia akina mama kupata huduma za ANC*  Delivery in a facility?  *Na pia huduma za uzazi?*   1. How do you think these barriers can be addressed?   *Je, kwa maoni yako, changamoto hizi zaweza tatuliwa vipi?* |
| **C. BELIEFS AND PERCEPTIONS /Imani na unavyoona** | |
| Beliefs around MNCH Care | 1. Why do you think women do not deliver in health facilities?   *Je, unafikiria ni sababu gani zinafanya akina mama wakose kwenda kwa kituo cha afya kuzaa?*  Are there any social reasons?  *Kuna sababu za kitamaduni?*   1. Are there differences between younger women (adolescents) and older women (or those that have multiple children)? What are these?   *Je kuna utofauti wa wanawake wachanga na walio wazee (ama wale walio zaa watoto kadhaa)?* **Probe respondent for consideration around differences in age, disability, number and age of children, ethnicity and religion, level of wealth and education** |
| What are the beliefs and social norms around family planning and its use? In relation to spacing of children | 1. What are the beliefs around family planning in this community?   *Ni fikra gani kuhusu upangaji wa uzazi ziko kwenye jamii hii?*   1. As a health worker, what messaging do you provide around family planning?   *Kama kiongozi, ni jumbe gani unazopeana kwa watu kuhusu upangaji wa uzazi katika jamii hii?*   1. What about the beliefs around when to have children and how often?   *Je kuhusu fikra za ni wakati gani wa kupata watoto na baada ya muda gani?* |
| Beliefs around Breastfeeding | 1. In your experience as a CHW, what beliefs do women have around breastfeeding their newborns?   *Kwa maoni yako kama CHWni fikra gani wamama wanzo kuhusu kunyonyesha watoto wao?*  Children under 6 months?  Watoto walio na umri wa chini ya miezi 6? |
|  | 1. Can you tell me a little about female genital cutting in this community?   *Ebu nieleze kuhusu masuala ya kupasha tohara kwa watoto wa kike kwenye jamii hii?*   1. In your opinion, what are the reasons for this?   *Kwa maoni yako unaona ni kwa nini?*  As a community health worker, what are some of the messages you provide around this?  *Kama CHW ni ujumbe gani unapeana kwa kijiji hiki kuhusu kupasha tohara?* |
| **D. PRACTICES /mazoezi** | |
| What is the practice of men in supporting access and use of MNCH services? (incl. accompaniment). | I would like to ask about men’s roles in this community when it comes to supporting access to health services for women during pregnancy and delivery  *Je, jukumu la wanaume katika jamii hii kwenye maswala ya kupokea huduma za afya wakati wa uja uzito na kujifungua kwa kina mama ni lipi?*   1. How do husbands or male family members generally support women in accessing ANC/Delivery/PNC?   *Je, mabwana ama waume wanasaidia vipi wanawake ili waweze kufikia huduma za afya wakati wakati wa uja uzito, kujifungua, na huduma za baada ya kujifungua?*   1. Are men encouraged to play this role?   *Je, wanaume wanahimiwa wafanye hivyo?*  If yes, how?  *Kama ni kweli, kivipi?*  If no, why not?  *Kama hawaambiwi, kwanini?*   1. What would prevent men from playing an active role in supporting women’s health issues?   *Kinacho zuilia wanaume wasisaidie katika masuala ya afya ya wanawake ni nini?*   1. What are some of the rules of the facility around this?   *Ni sheria gani ziko katika kituo cha afya kuhusu mambo haya?* |
| Health Worker Treatment | 1. In your experience, how do male (and then for female) clients treat you? *Kwa maoni yako, je wateja wakiume ama wakike wanakuchulia vipi?* |
| Exclusive Breastfeeding / Kunyonyesha mtoto pekee kwa miezi 6 | 1. What are the customs around breastfeeding in this community   *Ni itikadi gani/mila kuhusu kunyonyesha watoto ziko katika jamii hii?*   1. When a woman delivers in the facility, what is she instructed to do in terms of feeding her newborn?   *Mwanamke akizaa kwa kituo cha afya, anapewa maelekezo gani kuhusiana na lishe ya mtoto aliyezaliwa?* |
| **E. POLICIES, RULES/ sera na sheria** | |
| Health Facility Governance uongozi/utawala wa kituo cha afya | 1. What are the tasks performed by male and female CHWs?   *Ni majukumu gani yanayotekelezwa na CHW wakiume na wakike?*  Is there a difference between those performed by men and those by women?  *Je, kuna tofauti kati ya majukumu yanayotekelezwa na CHW wa kiume na wa kike?* |

**KII –Ministry of Health Representative**

| **INTRODUCTION** | |
| --- | --- |
| Introduction | Thank you for meeting with me today. I would like to start with getting to know you a little better. Could you tell me how long you have worked at the Ministry of Health and what is your role? |
| **A. ACCESS TO HEALTH RESOURCES /upatikanaji wa huduma za afya** | |
| Availability of Services | 1. To start, can you tell me about some of the MNCH services that are available at various facilities? 2. Are there services not provided that should be?   If yes, what are they?  Are there needs not covered?  If yes, what are they? |
| Challenges/Barriers | 1. In your opinion, what are the main challenges that women of reproductive age face in accessing MNCH care? Are there differences for adolescents? 2. What barriers do you see for women in attending ANC services?   For delivery in a facility?  For PNC?  **Probe respondent for consideration around differences in age, disability, number and age of children, ethnicity and religion, level of wealth and education**   1. How do you think these barriers can be addressed? |
| **C. BELIEFS AND PERCEPTIONS IMANI NA UNAVYOONA** | |
| Beliefs around MNCH Care | 1. What do you think some of the reasons why women do not deliver in health facilities?   Are there any social reasons?   1. Are there differences between younger women (adolescents) and older women (or those that have multiple children)? What are these? |
| **D. PRACTICES** | |
| What is the practice of men in supporting access and use of MNCH services? (incl. accompaniment) | Now, I would like to ask about men’s roles in supporting access to health services for women during pregnancy and delivery.   1. Do husbands or male family members generally accompany their wives/female family members to ANC visits?   For delivery?  For PNC?  Why or Why not?   1. What are some of the rules of the facility around this? 2. What are some of the MoH directives/messaging around this? |
| **E. POLICIES, RULES** | |
| Rules/Policies | Now, I would like to ask about some of the MoH strategies, policies and directives around MNCH.   1. What are some of the key strategies in place around MNCH? Ni mikakati hiyo mibomu vino kuhusu MNCH? 2. What are some of the policies and directives around Family Planning provided by the Ministry of Health?   What about messaging around number of children and spacing of children? Are there specific policies in regards to who can access services? Adolescents? Poor? Disabled?  **Probe respondent for consideration around differences in age, disability, number and age of children, ethnicity and religion, level of wealth and education** |
| Health Facility Governance | 1. Can you tell me a little about the governance structures at health facilities? Who sits on these committees?   What are their roles? |

**KII – Women’s Organization Representative**

| **INTRODUCTION** | |
| --- | --- |
| Introduction | Thank you for meeting with me today. I would like to start with getting to know you a little better. Could you tell me how long you have you lived in this community?  *Asante sana kwa kupatana na mimi leo. Ningependa tuanze kujuana kidogo. Umeishi kijiji hiki kwa muda gani?*  How long have you been in this organization?  *Umekuwa kwenye kikundi hiki kwa muda gani?*   1. What is the main focus of this organization   *Ni lengo gani kuu la hiki kikundi?*  What is your role?  *Una jukumu gani katika kikundi hiki?* |
| **A. ACCESS TO HEALTH RESOURCES/ upatikanaji wa rasilimali** | |
| Availability of Services/ | 1. How well do the current services available in this community respond to women’s needs?   *Je, huduma zinazopatikana hapa kijijini zinatosheleza mahitaji ya akina mama?*  **Probe respondents for consideration around differences in age, disability, number and age of children, ethnicity and religion, level of wealth and education**   1. Are there services not provided that should be?   *Kuna huduma ambazo ni muhimu na hazipatikani?*  If yes, what are they?  *Kama zipo, ni zipi?*  Are there needs not covered in this community?  *Kuna mahitaji ambayo hayaja angaziwa huduma hapa kijijini?*  If yes, what are they?  *Kama zipo, ni zipi?* |
| Challenges/Barriers | 1. In your opinion, what are the challenges that women in your community face in accessing MNCH care in the community?   *Kwa maoni yako, ni changamoto zipi ambazo wanawake wanapitia ili kufikia huduma za MNCH kwenye jamii hii?*   1. What barriers do you see for women in attending ANC services?   *Ni vizuizi gani ambavyo vinazuia akina mama kupata huduma za ANC*  Delivery in a facility?  *Na pia huduma za uzazi?*   1. How do you think these barriers can be addressed?   *Je, kwa maoni yako, changamoto hizi zaweza tatuliwa vipi?* |
| **B. DECISION MAKING/kufanya uamuzi** | |
| How can women’s participation in decision-making at the household and community levels be improved? | 1. In your opinion, what is the main role of women and men in the household? *Kwa maoni yako ni jukumu gani kubwa la wanawake na wanaume katika nyumba?*   What about in the community?  *Na je kwenye jamii nayo?*   1. When it comes to decision making in the household, who should be making decisions generally?   *Ikifika katika kufanya maamuzi kwenye nyumba, ni nani wa kufanya maamuzi kwa kawaida?*  What about when it comes to issues around pregnancy and delivery?  *Na ikiwa kwa masuala ya uja uzito na uzazi ni nani hufanya maamuzi?*   1. Are there things that your organization recommends to either men or women about this?   *Kuna mambo fulani ambayo shirika lako linapendekeza kwa wanaume ama wanawake kuhusu mambo haya?* |
| **C. BELIEFS AND PERCEPTIONS/Imani na unavyoona** | |
| Beliefs around MNCH Care | 1. Why do you think women do not deliver in health facilities?   *Je, unafikiria ni sababu gani zinafanya akina mama wakose kwenda kwa kituo cha afya kuzaa?*  Are there any social reasons?  *Kuna sababu za kitamaduni?*  Are there differences between younger women (adolescents) and older women (or those that have multiple children)? What are these?  *Je kuna utofauti wa wanawake wachanga na walio wazee (ama wale walio zaa watoto kadhaa)?* **Probe respondent for consideration around differences in age, disability, number and age of children, ethnicity and religion, level of wealth and education** |
| What are the beliefs and social norms around family planning and its use? In relation to spacing of children? | 1. What are the beliefs around family planning in this community?   *Ni fikra gani kuhusu upangaji wa uzazi ziko kwenye jamii hii?*  What messaging does your organization provide around family planning?  *Ni maelezo yapi kuhusu upangaji wa uzazi ambayo shirika lako hupeana?*  What about the beliefs around when to have children and how often?  *Je kuhusu fikra za ni wakati gani wa kupata watoto na baada ya muda gani?* |
| What are the social beliefs about age of marriage, FGM, and how are these beliefs or norms enforced? | 1. Could you tell me a little bit about marriage practices in the community?   *Unaweza kunieleza kidogo kuhusu masuala ya ndoa kwenye jamii hii?*  At what age do people generally get married?  *Watu huwa wanaoana wakiwa na umri gani?*  Is it the same for men and women?  *Na je umri huo ni sawa kwa wanawake na wanaume?*  Why? *Kwanini?*   1. What happens if someone outside of this age gets married (older or younger)?   *Kutafanyika nini kama mtu aliye nje ya umri huu akioa (awe ni mzee ama wa umri wa chini)*  What messaging does your organization provide around having and spacing children? Ni ujumbe upe shirika lako lapeana kuhusu kupata watoto na upangaji wa uzazi?   1. What are some of the main practices around female genital cutting in this community?   *Huwa mnafanya nini kuhusu masuala ya kupasha tohara kwa watoto wa kike kwenye jamii hii?*  In your opinion, what are the reasons for this?  *Kwa maoni yako unaona ni kwa nini?* |
| **D. PRACTICES/mazoezi** | |
| Exclusive Breastfeeding/ kunyonyesha mtoto kwa miezi 6 bila chengine chochote | 1. What are the customs around breastfeeding in this community?   *Ebu nieleze mila ama desturi kuhusu kunyonyesha mtoto kijiji hiki*   1. When a woman delivers in the facility, what does she normally do in terms of feeding her newborn?   *Mwanamke akizaa kwa kituo cha afya, kawaida huwa anafanya nini ki masuala ya lishe ya aliyezaliwa?* |
| What is the perceived role of men in supporting access and use of MNCH services? (incl. accompaniment) | I would like to ask about men’s roles in this community when it comes to supporting access to health services for women during pregnancy and delivery  *Ningependa kuuliza kuhusu jukumu la wanaume katika jamii hii kwenye maswala ya kupokea huduma za afya wakati wa uja uzito na kujifungua kwa kina mama ni lipi*   1. How do husbands or male family members generally support women in accessing ANC/Delivery/PNC?   *Je, mabwana ama waume wanasaidia vipi wanawake ili waweze kufikia huduma za afya wakati wakati wa uja uzito, kujifungua, na huduma za baada ya kujifungua?*   1. Are men encouraged to play this role?   *Je, wanaume wanahimiwa wafanye hivyo?*  If yes, how?  *Kama ni kweli, kivipi?*  If no, why not?  *Kama hawaambiwi, kwanini?* |

**Closing all sessions:**

1. Thank respondent
2. Remind respondent of the information letter and how they can find out more or ask any questions
3. Remind the respondent that their answers will remain confidential
4. Gather all notes and materials

| **AGA KHAN FOUNDATION, EAST AFRICA/AGA KHAN UNIVERSITY (EAST AFRICA)/AGA KHAN HEALTH SERVICES** |
| --- |
|  |

| **Access to Quality Care through Extending and Strengthening Health Systems (AQCESS): Gender assessment of maternal, neonatal and child health indicators in Kaloleni and Bomachoge-Borabu sub-counties in Kenya. Upatadzi wa huduma bora kukirira upanuzi na kuimarisha mifumo ya afya (AQCESS) tathmini ya vifaa, neonatal na viashiria zha afya ya ahoho kaunti thithe za Kaloleni na Bomachoge-Borabu Kenya. Upatikanaji wa huduma bora kupitia upanuzi na kuimarisha mifumo ya afya (AQCESS) tathmini ya vifaa, watoto wachanga wa chini ya mwezi mmoja na viashiria vya afya vya watoto kaunti ndogo za Kaloleni na Bomachoge-Borabu Kenya.** |
| --- |
|  |
|  |

Hello, my name is ________________________________________________ and I am working with the Aga Khan Development network who have partnered with the Ministry of Health of the government of Kenya to conduct a gender assessment study at the community level for maternal, neonatal and child indicators of this community in Kaloleni, Rabai and Bomachoge sub county **[adopt as necessary].** I am going to give you information and request you to be part of this survey. We would very much appreciate your participation. Kunautu dzina rangu ni ……………………..na nahenda kazi na AGA KHAN development network ambao mana shirikiana na wizara ya afya ya serikali ya kenya kuhenda tathmini ya jinsia kwa kijijini kuhusu mautu ga uzhazi, ahoho atsanga amwezi mwenga na viashiria zha ahoho a kijiji kaunti thithe za Kaloleni, Rabai na Bomachoge (kiriza ikidimikika) ndakupa maelezo na navoya ukale naswi kwa uu utafiti. Fundashukuru sana kwa ushirikiano wako. Jambo, jina langu ni ……………..na nafanya kazi na AGA KHAN development network ambao wana shirikiana na wizara ya afya ya serikali ya Kenya kufanya tathmini ya jinsia kijijini kuhusu masuala ya uzazi, watoto wachanga wa chini ya mwezi mmoja na viashiria vya watoto walio kijijini kaunti ndogo za kaloleni Rabai na Bomachoge **(pitisha ikiwezekana)** ntakupa maelezo na naomba uweze kuwa nasi katika utafiti huu.

**Purpose /Madhumuni**

The overall objective of the gender assessment is intended to provide AQCESS project staff with detailed information about the key gender issues within the context of the two project sub-county implementation areas of Kaloleni and Kisii, and related to the project interventions. Specifically, a focus on gender equality examines how differences in power relations result in differential risks, vulnerabilities, and outcomes in health for men and women / kwa jumula lengo ra tathmini ya jinsia ni kulazha ahendakazi a mradi wa AQCESS na habari kuhusu na mautu mabomu ga jinsia kwa mazingira ga miradi miri ya kaunti thithe katika utekelezaji Kaloleni na Kisii. Na mautu ganago husiana na hathua za miradi. Sanasana lengo ni usawa wa jinsia kaunyesa tofauthi kuhusiana kwa nguvu na hathari za utofauti,unyonge na matokeo ga afya ya alume na ana ache.

**Participant selection utsaguzi wa ashiriki/ uchaguzi wa washiriki**

To take part in the current assessment you must be a resident of Kaloleni, Rabai and Bomachoge Borabu sub-counties. You have been selected as a respondent because you fit the selection criteria. Ili kupatha nafasi kwa tathmini ii inayoenderera lazima ukale mkaladzi wa kaunti thithe za kaloleni, rabai na Bomachoge Borabu. Udzatsagulwa here mhojiwa sababu unathosha kwa maegezo ga utsaguzi. Ili kupata nafasi kwa tathmini hii inayoenderera lazima uwe mkaazi wa kaunti thithe za Kaloleni, Rabai, na Bomachoge Borabu, umechaguliwa kama mhojiwa sababu unatosha kwa maegezo ya uchaguzi.

**Procedures / tarathibu/ taratibu**

The discussion/interview will take about one hour and will include questions about your community, your health and the health of children under 5 years of age and gender issues. Masumuriro gaga gandahala here saa mwenga, maswali ganda husu atu kuku midzini na afya yakwako na afya ya ahoho mario tsini ya miaka mitsano na mambo ga jinsia. Mazungumzo /mahojiano haya yatachukua takriban saa moja pamoja na maswali kuhusu jamii, afya yako na afya ya watoto walio na umri chini ya miaka mitano, na maswala ya jinsia.

**Risks and discomforts hathari na kubujwa/ hatari na kusumbuliwa**

The interview has no risks to you or your health. However, if a question causes any anxiety or discomfort you may choose not to answer without giving a reason and we will proceed to the next question. Mahojiano gaga kagana hathari kwako na kwa afya yako. Lakini kala swali rorosi rindakupa wasiwasi ama kubujwa unadima kukahala kujibu bila kulazha sababu na fundaenderera na swali ringine ,/ mahojiano haya hayana hatari kwako na kwa afya yako, lakini kama swali lolote litakupa wasiwasi ama kukusumbua unaweza kukataa kujibu bila kupeana sababu na tutaendelea na swali lengine.

**Benefits /faidha/ faida**

The findings from the gender assessment for the AQCESS project which will be based on your participation will inform the activities and interventions that we plan to implement in this area. These activities are aimed at improving the services given by the sub county department of health to mothers, newborns and children under the age of 5 years in order to improve their health. Matokeo kumbola kwa tathmini ii ya mradi wa AQCESS indategemea na ushirikiano undafupa habari kuhusu shughuli na kungirirana na mpango wakutekeleza hathu haha. Shughuli zizi zina lenga kuboresha huduma zinazobozwa ni kaunti thithe za idara ya afya kwa ani mahma, madzio zhala na ahoho Mario tsini ya miaka mitsano ili kuboresha afya zao / matokeo ya kutoka kwa tathmini hii ya mradi wa AQCESS itategemea na ushirikiano, utatupatia habari kuhusu shughuli na kuingililiana na mpango wakutekeleza mahali hapa. Shughuli hizi zina lenga kuboresha huduma zinazotolewa na idara za afya za kaunti ndogo kwa akina mama walio zaa na watoto walio na umri wa chini ya miaka mitano.

**Confidentiality/ usiri/usiri**

All information collected will be kept confidential. The completed and signed Consent Form will be placed together. All research materials will be given a confidential number that will be known only by a few people involved in this research. All research materials will be assigned a confidential number for coding purposes. Results will not be released or reported in any way that might allow for identification of individual participants. All information will be aggregated and will be used only for community reference and not for individuals. Your name will not be associated with the findings. Maelezo gosi kumbola kwa uthafiti uu ganadikwa siri. uthafiti udziogonywa na kusianiwa fomu ya makubaliano indakiwa hamwenga. Vifaa zhosi zha uthafiti vindapewa usiri msthari wa mbere vindmanyikana na atu achache ambao madzahusika na uthafiti uu. Matokeo ga uthafiti kaundambozwa ama kuripotiwa kwa ngira yoyosi ambayo indahenda ashiriki kumanyikana na atu ambao kamahusika na uthafiti uu. Vifaa zhosi zha uthafiti vindapewa usiri kwa mchakato wa coding. Matokeo kaganda mbozwa ama kuripotiwa kwa ngira yoyosi ambayo indahenda umanyikane. Maelezo gosi ganda pimwa na kuhumika hakeye kwa kumbu kumbu za kijiji na si kwa atu kibinafsi. Dzinaro karindahusiana na matokeo ga uthafiti uu / maelezo yote kutoka kwa utafiti huu yatawekwa siri. Utafiti ulioisha na fomu za rithaa zilizosainiwa zitawekwa pamoja. Vifaa vyote vya utafiti vitapewa usiri mstari wa mbele na vitajulikana na watu wachache ambao wanahusika na utafiti huu. Matokeo ya utafiti hayatatolewa ama kuripotiwa kwa njia yoyote ambayo itafanya washiriki kujulikana na watu ambao hawahusiki na utafiti huu. Vifaa vyote vya utafiti vitapewa usiri kwa kutumia nambari maalum. Matokeo hayatatolewa ama kuripotiwa kwa njia yoyote ambayo itafanya ujulikane. Matokeo yote yatapimwa na kutumika kipekee kwa kumbukumbu za kijiji na si kwa watu binafsi. Jina lako halitahusiana na matokeo ya utafiti huu.

**Voluntary Participation ushiri wa hiari /ushiriki wa hiari**

All participation in this research is voluntary. You are free to decide if you want to take part or not. If you do agree to take part now, you can change your mind at any time during the interview without any implications. But we hope you will participate to the end. Kudziunga na uthafiti uu ni kwa hiari. Una uhuru wa kuhenda uamuzi kala udahenza kujumuika naswi ama kwenzi. Kala undakubali kujumuika naswi vikara, unadima kubadilisha mawazogo wakathi wowosi. Wakathi wa mahojiano unadima kubadilisha uamzi wako bila matukio gogosi gando kudhuru. Funa mathumaini undashirikiana naswi tha mwisho. Kushiri kwenye utafiti huu ni kwa hiari. Una uhuru wa kufanya uamuzi kama ungependa kujumuika nasi ama la. Kama utakubali kujumuika nasi sasa, unaweza kubadilisha mawazo yako wakati wowote wa mahojiano bila matokeo yoyote mabaya kwako. Tuna matumaini utashirikiana nasi hadi kikomo.

**Contact information maaelezo ga mawasiliano/ maelezo ya mawasiliano**

For more information about this study, you can contact the researchers who are responsible: Ms. Angela Ngetich (+254 780660080) or Lucy Nyaga (041 2226950), Kennedy Mulama (0726741499 ) Kwa maelezo kuhusu uthafiti uu, unadima kuwasiliana na athafiti manaohusika. Bi .Angela Ngetich (+254 780660080) or Lucy Nyaga (0412226950), Kennedy Mulama (0726741499). Kwa maelezo kuhusu utafiti unaweza kuwasiliana na watafiti wanaohusika. Bi .Angela Ngetich (+254 780660080) or Lucy Nyaga (041 2226950), Kennedy Mulama (0726741499).

**Consent Statement Maelezo ya makubaliano ya kushiriki kwenye utafiti**

I have read the preceding information, or it has been read to me. I have had the opportunity to ask questions about it and they have been answered to my satisfaction. I consent voluntarily to be a participant in this study and understand that I have the right to withdraw from the interview/discussion at any time. Nidzashoma maelezo gadzigo thangulia, ama nidzashomerwa maelezo gaga. Nidzapatha nafasi ya kuuza maswali na nikajibiwa tototo sana. Na rithaa kwa hiari kukala ndashirikiana kwa uthafiti na naelewa ukala nina uhuru wa kukahala kuenderera na mahojiano/mazungumzo wakathi wowosi. Nimesoma maelezo yaliyo tangulia ama nimesomewa maelezo haya, nimepata nafasi ya kuuliza maswali na nikajibiwa vyema.na nimekubali kwa hiari kuwa nitashiriki kwa utafiti na naelewa kwamba nina uhuru wa kukataa kuendelea na mahojiano/mazungumzo.

**🞎** I agree to be interviewed (*please tick*) nidzakubali kushiriki (tafadhali ika tiki) kama nimekubali kushiriki kwenye utafiti huu (tafadhali weka tiki)

**🞎** I do not agree to be interviewed (*please tick*) senzi kushiriki (tafadhali ika tiki) kama sijakubali kushiriki ( tafadhali weka tiki)

**Respondents Signature: Saini ya mshiriki/ Saini ya mshiriki**

______________________________ **Date: Tharehe/Tarehe**_____________________

**Thumbprint of the respondent named above if they cannot write: alama ya gumba kala mhojiwa kamanya kundika alama ya gumba kama mhojiwa hajui kuandika**

_____________________

I, the undersigned, have fully explained the relevant details of this survey to the respondent to consent. Mimi nidziye saini nidzaeleza maelezo muhimu kwa uthafiti uu kwa mhojiwa/ mimi niliye saini nitaeleza maelezo muhimu kwa utafiti huu kama mhojiwa.

**Enumerator’s Name: Dzina ra Enumerator** /Jina la Enumerator_____________________

**Enumerator’s Signature: Saini ya Enumerator** /Saini ya Enumerator_________________

**Date/Tharehe/Tarehe:** ____________________

**For adolescent mothers, a guardian/parent/CHV signs here:**

**Name:** ____________________________________

**Signature:** _________________________________

**Date:** _____________________________________
