# Supplementary material for: Deliver on Your Own: Disrespectful Maternity Care in rural Kenya
Source: PLoS One. 2020 Jan 7;15(1):e0214836. doi: 10.1371/journal.pone.0214836 (PMC6946164; doi:10.1371/journal.pone.0214836)
Supplement: S2 Appendix — (DOCX) [file pone.0214836.s002.docx]

**Appendix 2: Kisii (Bomachoge Borabu) site – Study and consent documents (English & Gusii)**

**AQCESS Kenya Gender Assessment: FGD Guide for Community Health Committee Participant/ Omoroberio bwo amaboria abarengete ase ekomiti ye ekenyoro**

**Note to Facilitator:**

- Ensure participants meet the requirements in the recruitment form
- Follow the instructions indicated for each question
- Select a location that ensures privacy and space for participants to speak freely
- Welcome each participant as they arrive

**Introduction/Ase ogochakera:**

- Start the FGD by reading the informed consent statement outlining all aspects of the study
- Present the assessment and objectives of the FGD to all participants
- Obtain informed consent from participants, including consent for audio recording (if consent is not provided, do not audio record)
- Prior or after the FGD, ask participants to fill in the participant information form. During this time, snacks/beverages can be distributed
- Introduce the facilitator and note-taker and ask participants to introduce themselves
- Share information with participants about the use of the recorder and who will hear this information
- Conduct a short icebreaker to help participants feel comfortable

**Overall Note:** For all questions be sure to probe respondents for consideration around differences in age, disability, number and age of children, ethnicity and religion, level of wealth and education.

| **Reference Question** | **Key Questions/ Amaboria abene** |
| --- | --- |
| **A. ACCESS TO RESOURCES /Goikera enibo** | |
| Access to Services */Goikera obokoreri* | 1. Tell me a little bit about what the CHC does   *Intebie eki ekomiti ya abanyagetari be binyoro bagokora*  What is your role on this committee?  *Ensemo yao nerei ase ekomiti?*   1. What MNCH services are available to women in your community   *Mbokoreri ki bore aroro bwa abangina bebwateraneti?*   1. What types of services do they normally use?   *Mbokoreri ki bakoinya korwa*   1. Are there services needed that are not available   *Obokoreri mboroo mboganeiri iga botaiyio?*  If yes, what are they and why do you think they are not available  *Onye nabo*, *mborobi obo naende inki gekogera botaiyio?*   1. Are services available to all women (adolescents, disabled women, rich/poor etc)?   *Obokoreri imborure gochia ase abasubati bonsi (abasae, abasubati baremarete, abanda /abataka*, etc)?  Why?  *Aseki?*  Why not  *Inki gekogera?* |
| Barriers to Access */Ebitango bioogotaikera obokoreri* | 1. What are some of the challenges that you think women have in accessing services while they are pregnant?   *Mechando eki okorengereria ekobatanga abangina tibagoiikera obokoreri ekero bebwateranetie?*   1. Are there reasons women do not go to the health facility during pregnancy?   *Chisababu inchiroo chikoirera abasubati tibakogenda nyagetari ekero bebwateraneti?*  What about to deliver their child?  *Engaki rende ekero bagochia konyora omwana?*  If yes, what are they?  *Onye nabo imbiribi ebi?*   1. What about receiving services from Community health worker/community midwife?   *Inaki rende korwa ase CHW/CMW?*  Are there reasons women do not receive these services?  *Chisababu nchire oo chikogera abasubati batari konyora obokoreri?*   1. If women do receive services from CHW/CMW are there challenges in benefitting from the services?   *Onyore abangina bakonyora obokoreri korwa asea CHW/CMW, bokongu bonde mbore bakonyora yagera tibakonyora obokoreri obo?*  If yes, what are these challenges?  *Mbokongu ki obo onye nabo?*   1. Are there some women in the community that might have a different experience either positive or negative?   *Basubati bande mbare ase ebinyoro igaa barabwo banyorire etabauti, ya obuya gose obobe?*  What barriers might they face in accessing services in a health facility  *Mbitango ki bakonyora ase ogoikera obokoreri bwa nyagitari?*  What would you suggest to make it easier?  *Inki orasegete ase okoyakora raisi?* (Probe around age, ethnic group disability/*Uunenkia gokorigereria emiaka, egesaku, oboremaru,* etc.) |
| Men’s Roles | 1. Generally, how are men involved during ANC, Delivery or PNC?   *Ingaki abasacha bairete ribaga ase amangana yokogenda ekiriniki ekero omosubati ebwateranetie gose ekero anyorire omwana?*   1. Do men ever accompany women to a health facility for ANC /Delivery/PNC?   *Abasacha nkobakoba bare abasubati babo gochia ekiriniki ekero bebwateranetie/bagochia koibora/banyorire omwana?*  If not, why not?  *Onye taribo, naseki?*   1. How do you think men could be supported to take a more active role in women’s pregnancy, delivery, neo-natal, postnatal care?   *Inaki okorengereria abasacha barasegetwe erio egere baire ensemo enene ekero omosubati ebwareranetie, ekero agochia konyora omwana na ekero anyorire omwana?* |
| **B. Decision Making** | |
| Decision Making Processes and CHC Role /*Okoamua amo na emeroberio ase ekamati ya abanyagetari be ekenyoro* | 1. Who generally makes the decision on whether or not to go for ANC in a typical household?   *Ingo okoinya gokwanera nga nere buya nere buya omosubati kogenda ekiriniki ekero ebwateranetie?*  What about to deliver or not deliver in a health facility?  *Inaki rende ase okiborera gose gotaiborera nyagetari?*   1. How does the CHC make decisions?   *Inaki ekomiti ya abanyagetari be ebinyoro ekoamua amangana ayeren gete?*   1. How does the CHC address issues about accessibility so that all women can access services at a facility with CHWs?   *Ingaki bono ekomiti ya abanyagetari be ebinyoro bagokwanera engana ya abangina konyora obokoreri bwa nyagetari amo na abanyagetari be ebinyoro?* |
| **C. Beliefs, Perceptions and Social Norms/ *amangana yo okwegena egekoro,ebirengererio bia abanto abanyene nachinchera abanto bamenyete chikororekana inga imbuya chire*** | |
| Beliefs and Social Norms/ *Amangana yo okwegena egekoro,ebirengererio bia abanto abanyene nachinchera abanto bamenyete chikororekana inga imbuya chire* | Now, I would like to explore some of the beliefs that people have around marriage, family and breastfeeding.  *Bono nigo indigetie omanyie igoro ya ebinto abanto begenete ase enyuomo, eamate, na okogogonkia omwana amabere angina.*   1. At what age do most men and women get married in this community?   *Miaka ki abasacha na abasubati bakonywomwa gose konywoma?* (Probe around laws/ *Rigereria mono igoro ya amachiko*)  What happens if someone outside of the age groups gets married (older or younger)?  *Inki gekoba ekero omonto otaraika emiaka ya okonywomwa anywomire gose anywomirwe (omonene ase emiaka gose omoke)?*   1. What about in terms of having children?   *Inaki rende ase okonyora kwa abana?*  What is the normal custom around the number of children and spacing of children?  *Inki egekoro kegoteba ase enamba ya abana na okobanga oroiboro?*  Who decides this generally?  *Nigo ase obwenge obwate okobua?*   1. What about if families do not want to have more children?   *Inaki rende onye efamiri tetageti komenta omwana?*  What are the beliefs around use of contraception?  *Inki abanto begenete igoro ya amariogo yokobanga oroiboro*? (Probe around community leaders messaging around this*/ Uunengia inaki abarai bagokwanera engana eye*)   1. Now, I would like to ask about circumcision, or cutting of females   *Bono tiga imborie igoro okwaroka gose gosara kwa abana abanyaroka.*  What is the practice in this community?  *Inki abanto bainyete gokora ase ebinyoro?*  What are the reasons why it is practiced?  *Sababu ki bakorwa ase ogokora igo?*   1. If a woman is circumcised (cut), does this affect where she gives birth?   *Ogosarwa kwo omosubati ngoirera kore ogochora ase agochia koborera omwana?*   1. Do you think this or other practices may cause women harm?   *Norengereretie nanyare koretera omosubati bokongu bonde?*  What are they?  *Mborobi obo?*  Why or why not?  *Naseki gose?*   1. What is said in the community about the care a woman should receive during pregnancy, delivery and post-partum recovery?   *Inaki abanto bagokwana ase oborendi bokorwegwa gochia ase omosubati ekero ebwateraneti, akoibora gose anyorire omwana?*   1. What are babies (0-6 months) normally fed in this community?   *Inki abana be emetienyi 0-6 bakoragerigwa ase ebinyoro*?  Is there a reason for this?  *Sababu ki ekogera bakoragerigwa iga?*  If yes, what is the reason?  *Inki gose onye nabo?* |
| **D. Practices and Participation** | |
| CHC, Leadership and Governance/ *Oborai bwe ekomiti ya abanyagetari be ekonyoro* | I’d like to ask you a few questions about the roles and responsibilities of health service providers and health committees  */Bono ndigetie inkoborie make ya emeremo ya abanyagetari be ebinyoro na ekomiti*   1. What do community leaders say about women’s participation in government and community structures?   *Ninki abarai bagokwanera igoro ya abasubati konyorerwa ribaga ase oborai bwe esirikari na ebinyoro?*   1. What about on this committee, how do you think you are perceived by the community   *Igaki rende ase ekomiti eye oroche ekenyoro gekobaira?*   1. How do women and men become members of this committee?   *Inaki abasacha na abasubati bagosoa ase ekomiti eye?*  What kind of experience do they require?  *Mbomanyi ki botakeire?*   1. How do you think women’s participation in community health structures could be improved?   *Inaki orengereretie abasubati barasegetwe konyorerwa ribaga ase oborai bwe ebinyoro?* |
| **E. Policies, Rules and Program Messaging** | |
| Health Messaging | Finally, I would like to ask you about some of the messages that are distributed around health promotion activities in your area  *Omoerio nindigetie inkoborie igoro ya amangana aria akogenda ase ebinyoro akoraria igoro ya gwetenenera na koira ensemo ase okorigereria afia yao.*   1. Have you heard health related messages on the radio, TV or in the community?   *Inee kwanya koigwa oboraria bwa amangana a fia ase eretio, etibi gose ase ekenyoro?*   1. Where did you hear these messages?   *Ngai kwaigwete mangana ayai?* (Probe for television, radio, posters/print or community/*uunengia igoro ye etibi,eretio,chiposita gose ase ekenyoro)*   1. What kind of messages are being promoted?   *Mangana ki akorarigwa mono?*   1. Who were these messages targeting?   *Amangana mono ningo arengete?*  Could you relate to the messages?  *Nonyatebe inga amangana ayio imbuya are?* |
| Rules and Policies  *Amachiko na amarago* | 1. What are some of the rules or policies around MNCH services at health facilities?   *Marago ki gose imborai ki borure bwo obokoreri ase abasubati na abana babo?*   1. Do these make it easier or more difficult to access or use MNCH services   *Inee aya nigo ayakorire akongu goikera obokoreri bwa nyagetari gochia ase abasubati na abana babo?*  Explain  *Yeerere*. |

**Closing:**

1. Thank all participants
2. Remind participants of the information letter and how they can find out more or ask any questions
3. Gather all notes and materials

**AQCESS Kenya Gender Assessment: FGD Guide for Female Participants/Omoongoso gochia ase abasubati**

**Note to Facilitator**

- Ensure participants meet the requirements in the recruitment form
- Follow the instructions indicated for each question
- Select a location that ensures privacy and space for participants to speak freely
- Welcome each participant as they arrive

**Introduction / Ase ogochakera** **:**

- Start the FGD by reading the informed consent statement outlining all aspects of the study
- Present the assessment and objectives of the FGD for all participants
- Obtain informed consent from participants, including consent for audio recording (if consent is not provided, do not audio record)
- Prior or after the FGD, ask participants to fill in the participant information form (provide assistance as required). During this time, snacks/beverages can be distributed
- Introduce the facilitator and note-taker and ask participants to introduce themselves
- Share information with participants about the use of the recorder and who will hear this information
- Conduct a short icebreaker to help participants feel comfortable

**Overall Note:** For all questions be sure to probe respondents for consideration around differences in age, disability, number and age of children, ethnicity and religion, level of wealth and education.

| **Reference Question** | **Key Questions** |
| --- | --- |
| **A. ACCESS TO RESOURCES** | |
| Access to Services/ *Ogoikera obokoreri* | I would like to ask you about your experience accessing and using health services related to MNCH care  *Bono nigo ndigetie imanye make igo ya ayoomanyete igoro yo okonyora obokoreri bwa abasubati na abana babo ase chinyagetari.*   1. Can you describe your recent experience in going to a health facility in or near this community?   *Nonyare gokwanera kende ase aya gwaeteire ase nyagetari gose ange na ekenyoro kere ange naye?*   1. What services are available there?   *Mbokoreri ki bokoenanwa aroro?*  What services are not available?  *Mbokoreri ki botaiyio?*  **Adolescent Group** /abasae: Are you able to access all the services, even if you are younger?  *Konyora ore obokereri bwonsi nonya kore omosae?*   1. What about accessing services from community health workers?   *Ingaki rende konyora obokoreri korwa ase abanyagetari ba ebinyoro?*  What kind of services do you receive from them?  *Mbokoreriki okonyora korwa ase bare?*  How often?  *Ara karenga?* |
| Barriers to Access *Ebitango gochia ase ogoikera*  Practices and Participation | 1. What are some of the challenges you faced in getting checkups while you were pregnant?   *Mbitango ki kwaumeranete nabio ekero kware koringorigwa kore morito?*  What would you suggest to make it easier?  *Inki oratebe gekorwe erio egere ebe raisi?* (Probe for challenges /*Uunenkia ebitango*)   1. Who here delivered their child at a health facility?   *Ningo igaiga onyorerete omwana nyagitari*?  Can you tell me about this experience */Nonyare gontebia aya gwaeterete?*   - - Fees*/ Ogoakana*: Were the services free*/Obokoreri imbosa bwarwetwe*?   How did you pay for them*/ Ingaki gwaakanerete?*  What other costs did you incur/*Mbintoki binde kwagorete?*   - - Did you have to bring materials with you to the health facility/*Iyatakeire orete ebinto bionsi nyagitari?*   - Distance/*oboare:* How did you reach the facility */Inaki kwaigete nyagitari?* How long did it take you*/Chinsa irenga kwairete?*   - Staff/*ababwenia*: How did the staff treat you /*Inaki abagwenia bakoirete?* Did you receive services from a male or female provider/*Ingo okoete obogwenia nomonyagetari omsacha gose nomokungu?*   How did that make you feel/ *Inaki kwaigwete ase ayio?*   1. Would you go to the facility again to deliver   *Nogende naende koiborera nyagitari?*  Why or why not?  *Inki gose inki otachiere*? (Probe around staff, age, ethnic group, distance/ *Uunengia igoro ya abanyagitari ,emiaka,egesaku na oboare*)   1. Are there some women in the community that might have a different experience, either positive or negative?   *Basubati bande mbare ase ebinyoro igaa barabwo banyorire etabauti, ya obuya gose obobe?*  What barriers might they face in accessing services in a health facility  *Mmbitango ki biragere mbakonyora obokoreri bwa nyagetari?*  What would you suggest to make it easier  *Inki oratebe gekorwe egere ebe aororo?* (Probe around age, ethnic group, disability/ *uunengia emiaka* *egesaku oboremaru* etc.)   1. For those that did not deliver in a facility, what are some of the reasons why you did not go to a health facility to deliver?   *Ase baria batanyuorerete abana nyagetari,* e*sababu buna chirichi chiairerete gotanyorera omwana nyagetari*?  What prevented you from doing so?  *Inki kiare egetango?*   1. After birth, who here was checked by a health professional?   E*kero kwaiborete ingo igaiga monyagetari ki orange eoo?*  Where did this checkup take place?  *Ingai aya iga akoreretwe?* |
| **B. Decision Making** | |
| Decision Making Processes | 1. Who made the decision on whether or not to go for ANC   *Ningo oamuete kogenda gose gotagenda ekiriniki kia ANC?*  What about to deliver or not deliver in a health facility?  *Inaki rende koibora nyagetari gose koiborera inka?*   1. In which areas do you feel you can influence decisions in your household?   *Ensemo oroche oranyaregosegeta ase enka yao*? (Probe for age of marriage, nutrition, breastfeeding, seeking health care/ *Uunengia emiaka ya okonywooma endagera kogongia omwana okorigia oboringoria bwa nyagetari, etc*.)   1. What do you think would help in terms of increasing your ability to make more decisions about your own health?   *Inki okorengereria kerabe ekio omochango omonene ase aye omonyene kwenachera aya arengete afia yao?* |
| Male Support and Accompaniment | 1. Generally, how are men involved during ANC, Delivery or PNC /pregnancy?   *Inaki okorengereria abasacha barasegetwe erio egere baire ensemo enene ekero omosubati ebwateranetie?*   1. Who accompanied you to the health facility during your last pregnancy or to deliver in the facility? (probe if a male spouse or family member accompanied)   *Ingo ogokobete gochia ekiriniki engaki kwabwate oborito bwo omoerio gose ekero gwachiete koibora omwana?*   1. What other support does your spouse or male family member provide to help you access/use health services?   *Mbokonyi ki bonde kwanyorire koru as omogaka oo gose mosacha onde bwesi bwe efamili ekero ogochia nyagitari?*   1. How do you think men can be supported to take a more active role in women’s pregnancy or neo-natal care?   *Inaki orengereretie abasacha barasegetwe egere koira ensemo enene ekero omongina ebwateranetie gose omwana eken’gwerere akorendwa* |
| **C. Beliefs and Perceptions** | |
| Beliefs and Social Norms | 1. At what age do most men and women get married in this community?   *Miaka ki abasacha na abasubati bakonywomwa gose konywoma?*   1. What happens if someone outside of the age groups gets married (older or younger)?   *Inki gekoba ekero omonto otaraika emiaka ya okonywomwa anywomire gose anywomirwe* /*omonene ase emiaka gose omoke*)?   1. What about in terms of having children?   *Inaki rende ase okonyora kwa abana,*  What is the normal custom around the number of children and spacing of children?  *Inkie/gekoro kegoteba ase enamba ya abana?*  Who decides this generally?  *Nigo ase obwenge obwate okobua*?   1. What about if families do not want to have more children?   *Inaki rende onye efamiri tetageti konyora mwana*?  What are the beliefs around use of contraception?  *Inki abanto begenete igoro ya amariogo yookobanka oroiboro?* (Probe around community leaders messaging around this /*Uunengia inaki abarai bagokwanera engana eye)*   1. Now, I would like to ask about circumcision, or cutting of females   *Bono tiga imborie igoro okwaroka gose gosara kwa abana abanyaroka*.  What is the practice in this community?  *Inki abanto bainyete gokora ase ebinyoro??*  What are the reasons why it is practiced?  *Sababu ki bakorwa ase ogokora igo?*   1. If a woman is circumcised (cut), does this affect where she gives birth?   *Ogosarwa kwo omosubati ngoirera kore ogochora ase agochia koborera omwana?*   1. Do you think this or other practices may cause women harm?   *Norengereretie ogosaru koretere omosubati bokongu bonde?*  What are they?  *Mborobi obo?*  Why or why not?  *Naseki gose?* |
| **D. Practices and Participation** | |
| Breastfeeding Practices  + Norms on Breastfeeding | Who here has children under 6 months?  *Ning’o igaa obwate abana bare inse ye emitienyi 6?*  We are going to talk about feeding of these children  *Ngochia tore gokwanera igoro yo okorageria abana aba.*   1. What do you normally feed your children under 6 months?   *Inki moinyete koa abana baino bare inse ye emetienyi 6?*  Why?  *Naseki?*   1. Where did you first learn about the practices you described?   *Ngai ritangani kwamanyerete amangana aya?*  What are some of the challenges you have faced in breastfeeding your children?  *Inee mbokongu ki gweteire ase okomogongia omwana oo?*  Why do you think this is so?  *Inki okorengereria aya are iga?* (Probe for time to breastfeed, support from male family member, physical challenges / *Uunengia igoro ya chinkaki chio okomogonkia omwana*, *ogosirwa* *na onde ore omosacha ase enka*, */nobwate boremaru bonde)* |
| **E. Policies, Rules and Program Messaging** | |
| Health Messaging | Finally, I would like to ask you about some of the messages you have heard through health promotion activities in your area  *Omoerio nindigetie inkoborie igoro ya amangana aria akogenda ase ebinyoro akoraria igoro ya gwetenenera na koira ensemo ase okorigereria afia yao.*   1. Have you heard health related messages on the radio, TV or in the community   *Inee kwanya koigwa oboraria bwa amangana a fia ase eretio, etibi gose ase ekenyoro?*   1. What kind of messages did you hear?   *Mangana ki kwaigure mono?*   1. Who were these messages targeting?   *Amangana mono ningo arengete?*  Could you relate to the messages?  *Nonyatebe inga amangana ayio imbuya are?*   1. Where did you hear these messages   *Ngai kwaigwete mangana ayai?* (Probe for television, radio, posters/print or community*/uunengia igoro ye etibi,eretio,chiposita gose ase ekenyoro)* |
| Rules and Policies *amarago na oborai* | 1. What are some of the rules you know about at the health facility?   *Marago ki gose imborai ki borure bwo obokoreri ase abasubati na abana babo*?  Do these make it easier or more difficult to access or use MNCH services?  *Inee aya nigo ayakorire akongu goikera obokoreri bwa nyagetari gochia ase abasubati na abana babo?*  Explain  *Yeerere.* |

**Closing/ Omoerio:**

- Ask the participants if they have anything else they would like to contribute in terms of the discussion held/*Boria abasiriki gose kende ngereo baratake komanya mono goetera ase ebigambo ebi*
- Thank all participants for their time
- Remind participants that the information will remain anonymous and confidential and that they can find out more or ask any questions by calling the number on the information letter
- Gather all notes and materials and ensure they are properly labeled
- Stop the recording and ensure security of the file for translation/transcription

**AQCESS Kenya Gender Assessment: FGD Guide for Male Participants/ Omoroberio bwo amabopria abarengete ase ekomiti ye ekenyor**

**Note to Facilitator:**

- Ensure participants meet the requirements in the recruitment form
- Follow the instructions indicated for each question
- Select a location that ensures privacy and space for participants to speak freely
- Welcome each participant as they arrive

**Introduction: Ase ogochakera:**

- Start the FGD by reading the informed consent statement outlining all aspects of the study
- Present the assessment and objectives of the FGD for all participants
- Obtain informed consent from participants, including consent for audio recording (if consent is not provided, do not audio record)
- Prior or after the FGD, ask participants to fill in the participant information (provide assistance as required). During this time, snacks/beverages can be distributed
- Introduce the facilitator and note-taker and ask participants to introduce themselves
- Share information with participants about the use of the recorder and who will hear this information
- Conduct a short icebreaker to help participants feel comfortable

**Overall Note:** Be sure to probe respondents for consideration around differences in age, disability, number and age of children, ethnicity and religion, level of wealth and education.

| **Reference Question** | **Key Questions/ Amaboria abene** |
| --- | --- |
| **A. ACCESS TO RESOURCES goikera enibo** | |
| Access to Services  *Goikera obokoreri* | I would like to ask you about your experience accessing and using health services in your community   1. Can you describe your recent experience in going to a health facility in or near this community?   *Tebera buna kuiretwe nyagetari*   1. What services are available there? What services are not available?   *Mbiribi bigokorwa aroro*   1. What about accessing services from community health workers? What kind of services do you receive from them? How often?   *Ingaki rende konyora obokoreri kowa ase abanyagetari ba ekenyoro*   1. What about health care for your wives or female family members, what kind of services are available for them?   *Inaki rende ase abasubati gose ase abarugi baino* |
| Barriers to Access ebitango bioogotaikera obokoreri  Practices and Participation Ebikoro amo no obwamo | 1. What are some of the challenges your wife (or female family member) has faced in getting checkups during pregnancy?   *Mbokung’u ki omongina oo gose mosubati onde bwensi bwe efamili aeteire ekero agounenkigwa ekero ebwateranetie?*  What would you suggest to make it easier?  *Inki oratebe keragere ebe raisi*? (Probe for challenges at a facility and that from community health workers) *Mbokongu ki bagoetera*   1. Whose child here was delivered at a health facility?   *Ing’o igaiga onyorerete omwana nyagitari?*  Can you tell me about this experience?  *Ntebie Naki yare?*   - - Fees/ *Chibesa*: Were the services free?/ *Mbosa gwakorereretwe*?   How did you pay for them? /*Onye gwakanete, ingaki gwakanete*?  What other costs did you incur? /*Nainde ninki gwaakanete*?   - - Did you have to bring materials with you to the health facility?   *Binto binde mbire gwachete nabio nyagitari?*   - - Distance *obwaare*: How did you reach the facility?   *Ngaki gwaigete nyagitari?*  How long did it take you? *Ngaki ki kwa irete goika?*   - - Staff/ *Abakori egasi*: How did the staff treat you? *Naki bakwariganetie?* How did they treat your wife/partner? /*Naki baariganetie omurugi oo gose eamate yao?*   Did she receive services from a male or female provider?  *Ing’o omokererete imerimo? No omosubati gose omosacha*?  How did that make you feel? / *Naki kwa igwete?*   1. Would your wife go to the facility again for delivery of another child?   *Omurugi oo nairane nainde ase enyagitari eye konyorera omwana onde aroro?* Why or why not?  *Aseki / gose?* (Probe around staff /*abakori egasi*, age /*imiaka*, ethnic group /*egesaku*, distance/ *obwaare*)   1. Are there some families in the community that might have a different experience either positive or negative?   *Eamate yeino nereo ebwate obokoreri obwaao, bore obuya gose obobe?*  What barriers might they face in accessing services in a health facility  *Bitango gi baranyore ase obokoreri bwanyagitari?*  What would you suggest to make it easier?  *Inki oratebe kirakore imerimo ebe emiororo* (Probe around age /*emiaka*, ethnic group/ *egesaku,* disability/ *oborema*, etc. *binto binde*)   1. For those whose child was delivered at home, what are some of the reasons why you did not go to a clinic?   *Oria omwana oye anyoreretwe inka, ninki kiagerete ngwagenda nyagitari?*  What prevented you from doing so?  *Mbitangoki biagerete?* |
| **B. Decision Making** | |
| Decision Making Processes enchera yo oko amua | 1. In your family who made the decision on whether or not to go for checkups during your wife/partner/ female family member’s pregnancy?   *Ase efamili yeino, ing’o okoamua gose ogende, gose togenda nyagitari korigererigwa engaki / omorugi/ / omosani/ omosubati are no oborito?*  What about to deliver or not deliver in a health facility  *Inaki igoro yokonyorera gose gotanyorera omwana ase nyagitari?*  Why  *inki?* |
| Male Support and Accompaniment/  Obokonyi bwo omosacha na oyumorekomo | 1. During the pregnancy of your last child how were you involved?   *Engaki yooborito bwo omwana oo bwo omoerio, Ng’aki gwakonyote*? (Probe around ANC/ *kogenda eclinic*, Delivery/ *okoibora*, PNC /*okoira omwana na omurigu gose* )   1. Who accompanied your wife/partner / female family member to the health facility during her pregnancy or to deliver in the facility?   *Ing’o bachiete amo no omurugi oo/mosani/omosubati bwe efamili gochia nyagitari eng’aki yo oborito gose okonyorera omwana nyagitari?*  Why this person? *Aseki omonto oyoiga?*   1. What other things did you do to support your wife/partner/female family member during her pregnancy and after her pregnancy?   *Mbito ki binde gwakororete ase ogokonya omorugi oo/omosani /omosubati bwe efamili engaki yo oborito bwaye? Gose ekero aibore?*  Why? *Aseki?* (Probe around breastfeeding /*Ngaki yo omwana kogonga*)   1. Do you think men can take a more active role in womens’s pregnancy or neonatal care?   *No kagerete ing’a abasacha mbaire ensemo enene ase ogokonya abang’ina engaki yoo oborito gose okogonkia?*  Why? *Aseki eranyarekane*  Why not? Ase ki eta*nyarekane?* |
| **C. Beliefs and Perceptions /obwegenwa no oborigereria** | |
| Beliefs and Social Norms/ obwegenwa amo ne chinchera chieito | 1. At what age do most men and women get married in this community?   *Miaka ki abasacha na abasubati bakonywomwa gose konywoma ase egesaku eke?*   1. What happens if someone outside of the age groups gets married   *Inki gekoba ekero omonto otaraika emiaka ya okonywomwa anywomire gose anywomirwe* (older or younger /*omonene ase emiaka gose omoke*)?   1. What about in terms of having children?   *Inaki rende ase okonyora kwa abana?*  What is the normal custom around the number of children and spacing of children?  *Inki egekoro kegoteba ase enamba ya abana na okobanga oroiboro?*  Who decides this generally?  *Nigo ase obwenge obwate okobua?*   1. What about if families do not want to have more children?   *Inaki rende onye efamiri tetageti konyora abana?*  What are the beliefs around use of contraception?  *Inki abanto begenete igoro ya amariogo yookobanka oroiboro?* (Probe around community leaders messaging around this/ *Uunengia inaki abarai bagokwanera engana eye*)   1. Do you think there are any practices that may cause women harm?   Norengereretie nanyare koretera omosubati bokongu bonde?  What are they? *Mborobi obo?*  Why or why not? *Naseki gose* |
| **E. Policies, Rules and Program Messaging** | |
| Health Messaging /obochenu bwe ring’ana | Finally, I would like to ask you about some of the messages you have heard through health promotion activities in your area.  *Omoerio nindigetie inkoborie igoro ya amangana aria akogenda ase ebinyoro akoraria igoro ya gwetenenera na koira ensemo ase okorigereria afia yao.*   1. Have you heard health related messages on the radio, TV or in the community? *Inee kwanya koigwa oboraria bwa amangana a fia ase eretio, etibi gose ase ekenyoro?* 2. What kind of messages did you hear?   *Bosemia ki kwaigwete?*   1. Who were these messages targeting?   *Amangana mono ningo arengete?*  Could you relate to the messages?  *Nonyatebe inga amangana ayio imbuya are?*   1. Where did you hear these messages?   *Ngai kwaigwete mangana ayai (*Probe for television, radio, posters/print or community/ *uunengia igoro ye etibi,eretio,chiposita gose ase ekenyoro)* |
| Rules/and Policies amachiko na amarago | 1. What are some of the rules you know about at the facility?   *Imborai ki borure bwo obokoreri ase enyagitari*   1. 25 Do these make it easier or more difficult to access or use MNCH services?   *Inee aya nigo ayakorire akongu goikera obokoreri bwa nyagetari gochia ase abasubati na abana babo?*  Explain. *Yeerere* |

**Closing: Omoerio**

- Ask the participants if they have anything else they would like to contribute in terms of the discussion held/*Baborie bonsi gose kende inkere baramente*
- Thank all participants for their time /*Baakere buya mono bonsi ase chisa chiabo*
- Remind participants that the information will remain anonymous and confidential and that they can find out more or ask any questions by calling the number on the information letter/*Bainyorie bonsi amangana nabekwe*
- Gather all notes and materials and ensure they are properly labelled
- Stop the recording and ensure security of the file for translation/transcription

**KII – Health Facility Personnel**

**Note to Facilitator**

- Ensure focus is on building rapport with the interviewee
- Note down any probing questions that are asked

**Introduction:/ogochekera**

- Introduce yourself and Present the assessment and objectives of the KII
- Read the information letter and consent form
- Obtain informed consent from respondent including consent for audio recording (if consent is not provided/onye gwancherwa tigokoba, do not audio record
- Ask if the respondent has any questions before beginning the interview

| **INTRODUCTION** | |
| --- | --- |
| Introduction | Thank you for meeting with me today. I would like to start with getting to know you a little better. Could you tell me your name, how long you have worked at this health facility and what is your role? |
| **A. ACCESS TO HEALTH RESOURCES** | |
| Availability of Services | 1. To start, can you tell me about some of the MNCH services that are available at this facility? 2. How well do the current health services available at this clinic respond to women’s and men’s needs? 3. Are there services not provided that should be? If yes, what are they? Are there needs not covered in this community? If yes, what are they? |
| Challenges/Barriers | 1. In your opinion, what are the challenges that women in this community face in accessing MNCH care at the facility level? 2. What barriers do you see for women in attending ANC services? Delivery in a facility? 3. How do you think these barriers can be addressed? |
| **C. BELIEFS AND PERCEPTIONS** | |
| Beliefs around MNCH Care | 1. Why do you think women do not deliver in health facilities? Are there any social reasons? 2. Are there differences between younger women (adolescents) and older women (or those that have multiple children)? What are these? |
| What are the beliefs and social norms around family planning and its use? In relation to spacing of children? | 1. What are the beliefs around family planning in this community? 2. As a health professional, what messaging do you provide around family planning at the clinic? 3. What about the beliefs around when to have children and how often? |
| **D. PRACTICES** | |
| What is the practice of men in supporting access and use of MNCH services? (incl. accompaniment) | Now, I would like to ask about men’s roles in this community when it comes to supporting access to health services for women during pregnancy and delivery.   1. Do husbands or male family members generally accompany their wives/female family members to ANC visits? Delivery? PNC? Why or Why not? 2. What are some of the rules of the facility around this? |
| Health Personnel Treatment | 1. In your experience, how do male (and then for female) clients treat you? 2. What is considered respectful treatment of male and or female clients? |
| Exclusive Breastfeeding | 1. What are the customs around breastfeeding in this community? 2. When a woman delivers in the facility, what is she instructed to do in terms of feeding her newborn? |
| **E. POLICIES, RULES** | |
| Rules/Policies | 1. Are there specific policies at the facility in regards to who can access services? Adolescents? Poor? Disabled? 2. For staff and management, are there any policies related to safe working environments in this facility? What are they? 3. What about personal leave? How is this applied? |
| Health Facility Governance | 1. What are the tasks performed by male and female health providers? Is there a difference between those performed by men and those by women? 2. Tell me a little about the governance structures at these facilities? Who sits on these committees? What are their roles? |

**KII – Ministry of Health Representative**

**Note to Facilitator**

- Ensure focus is on building rapport with the interviewee
- Note down any probing questions that are asked

**Introduction:/ogochekera**

- Introduce yourself and Present the assessment and objectives of the KII
- Read the information letter and consent form
- Obtain informed consent from respondent including consent for audio recording (if consent is not provided/onye gwancherwa tigokoba, do not audio record
- Ask if the respondent has any questions before beginning the interview

| **INTRODUCTION** | |
| --- | --- |
| Introduction | Thank you for meeting with me today. I would like to start with getting to know you a little better. Could you tell me your name, how long have you worked at the Ministry of Health and what is your role? |
| **A. ACCESS TO HEALTH RESOURCES** | |
| Availability of Services | 1. To start, can you tell me about some of the MNCH services that are available at various facilities? 2. Are there services not provided that should be? If yes, what are they? Are there needs not covered? If yes, what are they? |
| Challenges/Barriers | 1. In your opinion, what are the main challenges that women of reproductive age face in accessing MNCH care? Are the differences for adolescents? 2. What barriers do you see for women in attending ANC services? Delivery in a facility? PNC? 3. How do you think these barriers can be addressed? |
| **C. BELIEFS AND PERCEPTIONS** | |
| Beliefs around MNCH Care | 1. What do you think some of the reasons why women do not deliver in health facilities? Are there any social reasons? 2. Are there differences between younger women (adolescents) and older women (or those that have multiple children)? What are these? |
| **D. PRACTICES** | |
| What is the practice of men in supporting access and use of MNCH services? (incl. accompaniment) | Now, I would like to ask about men’s roles in supporting access to health services for women during pregnancy and delivery.   1. Do husbands or male family members generally accompany their wives/female family members to ANC visits? Delivery? PNC? Why or Why not? 2. What are some of the rules of the facility around this? 3. What are some of the MoH directives/messaging around this? |
| **E. POLICIES, RULES** | |
| Rules/Policies | Now, I would like to ask about some of the MoH strategies, policies and directives around MNCH.   1. What are some of the key strategies in place around MNCH? 2. What are some of the policies and directives around Family Planning provided by the Ministry of Health? 3. What about messaging around number of children and spacing of children? 4. Are there specific policies in regards to who can access services? Adolescents? Poor? Disabled? |
| Health Facility Governance | 1. Can you tell me a little about the governance structures at health facilities? Who sits on these committees? What are their roles? |

**KII – Women’s Organization Representative**

**Note to Facilitator**

- Ensure focus is on building rapport with the interviewee
- Note down any probing questions that are asked

**Introduction:/ogochekera**

- Introduce yourself and Present the assessment and objectives of the KII
- Read the information letter and consent form
- Obtain informed consent from respondent including consent for audio recording (if consent is not provided/onye gwancherwa tigokoba, do not audio record
- Ask if the respondent has any questions before beginning the interview

| **INTRODUCTION** | |
| --- | --- |
| Introduction | Thank you for meeting with me today. I would like to start with getting to know you a little better. Could you tell me your name, how long have you lived in this community, how long you have been in this organization?   1. What is the main focus of this organization and what is your role? |
| **A. ACCESS TO HEALTH RESOURCES** | |
| Availability of Services | 1. How well do the current health services available in this community respond to women’s needs? 2. Are there services not provided that should be? If yes, what are they? Are there needs not covered in this community? If yes, what are they? |
| Challenges/Barriers | 1. In your opinion, what are the challenges that women in this community face in accessing MNCH care? 2. What barriers do you see for women in attending ANC services? Delivery in a facility? 3. How do you think these barriers can be addressed? |
| **B. DECISION MAKING** | |
| How can women’s participation in decision-making at the household and community levels be improved? | 1. In your opinion, what is the main role of women and men in the household? What about in the community? 2. When it comes to decision making in the household, who should be making decisions generally? What about when it comes to issues around pregnancy and delivery? 3. Are there things that your organization recommends to either men or women about this? |
| **C. BELIEFS AND PERCEPTIONS** | |
| Beliefs around MNCH Care | 1. Why do you think women do not deliver in health facilities? Are there any social reasons? 2. Are there differences between younger women (adolescents) and older women (or those that have multiple children)? What are these? |
| What are the beliefs and social norms around family planning and its use? In relation to spacing of children? | 1. What are the beliefs around family planning in this community? 2. What messaging does your organization provide around family planning? 3. What about the beliefs around when to have children and how often? |
| What are the social beliefs about age of marriage, FGM, and how are these beliefs or norms enforced? | 1. Could you tell me a little bit about marriage practices in the community? At what age do people generally get married? Is it the same for men and women? Why? 2. What happens if someone outside of this age gets married (older or younger)? 3. What messaging does your organization provide around having and spacing children? 4. What are some of the main practices around female genital cutting in this community? 5. In your opinion, what are the reasons for this? |
| **D. PRACTICES** | |
| Exclusive Breastfeeding | 1. What are the customs around breastfeeding in this community? 2. When a woman delivers in the facility, what does she normally do in terms of feeding her newborn? |
| What is the perceived role of men in supporting access and use of MNCH services? (incl. accompaniment) | I would like to ask about men’s roles in this community when it comes to supporting access to health services for women during pregnancy and delivery.   1. How do husbands or male family members generally support women in accessing ANC/Delivery/PNC? 2. Are men encouraged to play this role? If yes, how? If no, why not? 3. What would prevent men from playing an active role in supporting women’s health issues? |

**Closing:**

1. Thank respondent
2. Remind respondent of the information letter and how they can find out more or ask any questions
3. Remind the respondent that their answers will remain confidential
4. Gather all notes and materials

**KII – Religious Leader/Abarai be ekanisa**

**Note to Facilitator: Aya amorengete omwoorokia**

- Ensure focus is on building rapport with the interviewee/*Ekerenga nigo kere korosia oboamate obuya gochia ase omoiraneria amaswari*
- Note down any probing questions that are asked/*rika inse amaboria aime mono araborigwe*

**Introduction:/ogochekera**

- Introduce yourself and Present the assessment and objectives of the KII*/ teba amarieta ao na kwemanyekania gose koorokia ekerenka kia amangana aya*
- Read the information letter and consent form/*soma riuko naboigo yeeorokie ekaratasi eria ya okoboria gwancherwa*
- Obtain informed consent from respondent /*Nyora ogwancherwa korwa kera ase oyomo*, including consent for audio recording /*komenta na ogwancherwa guo okobwata amangana ayio ase evideo* (if consent is not provided/onye gwancherwa tigokoba, do not audio record/ *tobaisa koyabwata amangana aya ase ekanda)*
- Ask if the respondent has any questions before beginning the interview/*boria gose omoiraneria nabwate koboria konde otarachaka amaboria*

| **INTRODUCTION** | |
| --- | --- |
| Introduction */Omochakano* | Thank you for meeting with me today  *Naakire buya mono ase okoumerana nainche*.  I would like to start with getting to know you a little better  *Nanchire gokomanya buya ake.*  Could you tell me your name/nontebie erieta rio, how long have you lived in this community/  *Geka kengani inaki kwamenyire ase ekenyoro*  How long you have been the religious leader in this community  G*eka kengani inaki kwabeire omorai bwe ekenisa?*  What is the main religion practiced in this community  *Kanisa ki ebwate abanto abange ase ekenyoro?*  As a religious leader in this community what is your role?  *Buna omorai bwe ekanisa ase ekenyoro, egetari kiao nkereki?* |
| **B. DECISION MAKING** | |
| How can women’s participation in decision-making at the household and community  *inaki abasubati koba aba bene ase okoamua amangana ye enka na ekenyoro*  levels be improved*/Inaki abasubati koba aba bene ase okoamua amangana ye enka na ekenyoro?* | 1. In your opinion, what is the main role of women and men in the household? What about in the community   *Ase aye omonyene emeremo emenene ya abasubati nerei ase enka na ekenyoro?*   1. When it comes to decision making in the household/   *Ekero gio okoira atua ase enka?*  Who should be making decisions generally  *Ningo otakeiere goteba ekio gekoba?*  What about when it comes to issues around pregnancy and delivery/  *Inaki ekero yaikeire amangana koba morito na koibora?*   1. Are there things that you recommend as a religious leader to either men or women about this   *Binto mbiroo aroro orasegete gochia ase omosacha gose omokungu?* |
| **C. BELIEFS AND PERCEPTIONS** | |
| What are the beliefs and social norms around family planning and its use  *Mbitoki abanto begenete buna egekoro na enchera abanto bamenyete abwo kare korengana okobanga oroiboro?* | 1. What are the beliefs around family planning in this community   *Mbitoki bie egekoro abanto begenete korengana na okobanga oroiboro?*   1. As a religious leader what messaging do you provide around family planning?   *Buna omorai bwe ekanisa,mbosemia ki okoru igoro ya okobanga oroiboro?*   1. What about the beliefs around when to have children and how often   *Ingaki rende egekoro abanto begenete ekero gio okonyora abana na egakeka kena ingaki?*  In relation to spacing of children  *Koenana ribaga omwana gochia oyonde* ? |
| What are the social beliefs about age of marriage/ FGM  *mbintoki abanto begenete igoro ya ogosara abana abaiseke*, *emiaka yo okonywomu,*  and how are these beliefs or norms enforced/  *na egekoro eke ingaki bare gokora barora inga giaikeraniere*? | 1. Could you tell me a little bit about marriage practices in the community   *Nonyeresere ake igo naki abanto bainyete konywuomana ase ebenyoro?*  At what age do people generally get married  *Miaka engana inaki abanto bakoira bamanya konyuoma gose konyuomwa*?  Is it the same for men and women  *Erengaine eye abasacha na abakungu?*  Why/ *naseki*?   1. What happens if someone outside of this age gets married/   *Inki gekoba ekero oyore isiko ya emiaka eye akonyuomwa*  (Older or younger/ *omoke gose omonene*)?   1. As a religious leader/*buna omorai bwe ekanisa*,   What messaging do you provide around having and spacing children?  *Mangana ki okorandia igoro ya okobanga oroiboro*?   1. What are some of the main practices around female genital cutting in this community?   *Inee imbitoki bigokorwa ekero abana abaiseke bagosarwa?*   1. In your opinion, what are the reasons for this?   *Ase ebirengererio bio inki gekogera bagokora igo gose*? |
| What is the perceived role of men in supporting access and use of MNCH services  *inki gekageire koba ensemo ya abasacha ase ogosira goikera obokoreri abasubati na abana babo?* (incl. accompaniment *na binde bikobwatia*) | Finally/*omoerio*, I would like to ask about men’s roles in this community when it comes to supporting access to health services for women during pregnancy and delivery  *Nindigetie gokoboria make igoro ya nsemo ki abasacha bairete kobasira abasubati ekero bebwateranetie gose bagochia koibora.*   1. How do husbands or male family members generally support women in accessing ANC/Delivery/PNC?   *Inaki abasacha gose mosacha onde ore ase enka agokonya abasubati kogenda ekeriniki ekero bebwateranetie/bagochia koibora/gose nyuma baiboire*   1. Are men encouraged to play this role   *Inee abasacha ngosegetwa bare gokora aya?* If yes *onye nabo nchera ki eye*, how? If no *onye taribo inki gekogera,* why not?   1. What would prevent men from playing an active role in supporting women’s health issues   *Inki kere egetango gochia ase abaacha gotasira abasubati igoro ya nyagetari?* |

**KII – Community Leader/Elder and Local Government Leader/omorai bwe ekenyoro/omorai bwe egeserekari ase ekenyoro**

**Note to Facilitator:/aya amorengete omwoorokia**

- Ensure focus is on building rapport with the interviewee/*Ekerenga nigo kere korosia oboamate obuya gochia ase omoiraneria amaswari*
- Note down any probing questions that are asked/*rika inse amaboria aime mono araborigwe*

**Introduction:/Ogochekera**

- Introduce yourself and Present the assessment and objectives of the KII*/ teba amarieta ao na kwemanyekania gose koorokia ekerenka kia amangana aya*
- Read the information letter and consent form/*soma riuko naboigo yeeorokie ekaratasi eria ya okoboria gwancherwa*
- Obtain informed consent from respondent /*Nyora ogwancherwa korwa kera ase oyomo*, including consent for audio recording /*komenta na ogwancherwa guo okobwata amangana ayio ase evideo* (if consent is not provided/onye gwancherwa tigokoba, do not audio record/ *tobaisa koyabwata amangana aya ase ekanda)*
- Ask if the respondent has any questions before beginning the interview/*boria gose omoiraneria nabwate koboria konde otarachaka amaboria*

| **INTRODUCTION** | |
| --- | --- |
| Introduction  *Omochakano* | Thank you for meeting with me today/naakire buya mono ase okoumerana nainche. I would like to start with getting to know you a little better/nanchire gokomanya buya ake. Could you tell me your name, how long have you lived in this community  *Geka kengana inaki kwamenyire ase ekenyoro, how long you have been a leader in this community/geka kengana inaki kwabeire omorai bwe ekenisa?*   1. As a leader in this community what is your role?   *Aye buna omorai ensemo yao nerei?* |
| **B. DECISION MAKING** | |
| How can women’s participation in decision-making at the household and community levels be improved  *Ase ekenyoroinaki bono abangina barasegetwedase ogokora amaamusi are aye engecho ase ekenyoro ?* | 1. In your opinion, what is the main role of women and men in the household? *Ase amangana ao, emeremo emeneene ya abasacha na abangina nerei?*   What about in the community?  *Naki rend ease ekenyoro?*   1. When it comes to decision making in the household who should be making decisions generally   *Ekero yaikkeire ase ogokora amaamusi as ease enka ningo otakeire gokora amaamusi ayio?*   1. What about when it comes to issues around pregnancy and delivery   *Inaki rende ekero yaikeire amangana ya koba morito na gochia koibora?*   1. Are there things that you recommend as a leader to either men or women about this   *Binto mbiro oranyare gosegeta gochia ase abasacha na abangina aye buna omorai?* |
| **C. BELIEFS AND PERCEPTIONS** | |
| What are the beliefs and social norms around family planning and its use  *Mbitoki abanto begenete buna egekoro na enchera abanto bamenyete abwo kare korengana okobanga oroiboro?* | 1. What are the beliefs around family planning in this community   *Mbitoki bie egekoro abanto begenete korengana na okobanga oroiboro?*   1. As a leader/buna omorai, what messaging do you provide around family planning   *Mangana okwegeria igoro ya okobanga oroiboro?*   1. What about the beliefs around when to have children and how often/   *Inaki rende ekero giokobanga indi riokonyora abana na geka ke ngana ingaki?* |
| What are the social beliefs about age of marriage/ FGM  mbintoki abanto begenete igoro ya ogosara abana abaiseke, emiaka yo okonywomu,  and how are these beliefs or norms enforced/  na egekoro eke ingaki bare gokora barora inga giaikeraniere? | 1. Could you tell me a little bit about marriage practices in the community   *Nonyeresere ake igo naki abanto bainyete konywuomana ase ebenyoro?*  At what age do people generally get married  *Miaka engana inaki abanto bakoira bamanya konyuoma gose konyuomwa?*  Is it the same for men and women  *Miaka engana inaki abanto bakoira bamanya konyuoma gose konyuomwa?*  Why? *Naseki?*   1. What happens if someone outside of this age gets married   *Inki gekoba ekero oyore isiko ya emiaka eye akonyuomwa* (older or younger/*omoke gose omonene)?*   1. As a leader/buna omorai, what messaging do you provide around having and spacing children?   *Mbosemia ki okorwa bousikanete na okobanga oroiboro?*   1. What are some of the main practices around female genital cutting in this community   *Mbintoki bigokorwa ekero abana abaiseke bagosarwa ase ekenyoro ki?*   1. In your opinion, what are the reasons for this   *Ase amaoni ao sababu ki chikogera bagokora igo?* |
| What is the perceived role of men in supporting access and use of MNCH services  */Inki gekageire koba nakio abasacha batakeier ghokora ase okobasira abasubati na abana babo?* (incl. accompaniment/ amo na kobachiesia) | Finally/omoerio kegima , I would like to ask about men’s roles in this community when it comes to supporting access to health services for women during pregnancy and delivery  *Nindigeti nkoborie make igoro ya ensemo abasacha bairete ase ebeinyo kobakonya abasubati goikera obokoreri bwa nyagetari ekero bagochia koibora gose bare morito.*   1. How do husbands or male family members generally support women in accessing ANC/Delivery/PNC?   *Ingaki abasacha gose basacha bande ase enka bakobasira abangina ase ogoikeria obokoreri bwa kogenda kiriniki ekero be bwateraneti/bagochia koibora /nyuma baibore*   1. Are men encouraged to play this role   *Abasacha ngosegetwa bare gokora aya?*  If yes, how?  *Oye nabo, nchera ki eye?*  If no, why not *inki gekogera onye taribo inki gekogera*   1. What would prevent men from playing an active role in supporting women’s health issues? *Ninki gekobatanga abasacha gotaira ensemo ase okobasira abangina amangana anyagetari?* |

**KII – Community Health Worker/ Omonyagetari bwe ekenyoro**

**Note to Facilitator:/aya amorengete omwoorokia**

- Ensure focus is on building rapport with the interviewee/*Ekerenga nigo kere korosia oboamate obuya gochia ase omoiraneria amaswari*
- Note down any probing questions that are asked/*rika inse amaboria aime mono araborigwe*

**Introduction:Ogochekera**

- Introduce yourself and Present the assessment and objectives of the KII*/ teba amarieta ao na kwemanyekania gose koorokia ekerenka kia amangana aya*
- Read the information letter and consent form/*soma riuko naboigo yeeorokie ekaratasi eria ya okoboria gwancherwa*
- Obtain informed consent from respondent /*Nyora ogwancherwa korwa kera ase oyomo*, including consent for audio recording /*komenta na ogwancherwa guo okobwata amangana ayio ase evideo* (if consent is not provided/onye gwancherwa tigokoba, do not audio record/ *tobaisa koyabwata amangana aya ase ekanda)*
- Ask if the respondent has any questions before beginning the interview/*boria gose omoiraneria nabwate koboria konde otarachaka amaboria*

| **INTRODUCTION** | |
| --- | --- |
| Introduction  */Ogochakera* | Thank you for meeting with me today/naakire buya mono ase okoumerana nainche. I would like to start with getting to know you a little better  *Nanchire gokomanya buya ake.*  Could you tell me your name?  *Nonyare gontebia erieta riao,*  How long you have been a CHW and what is your role  *Egeka kengania ingaki kwabeire omonyagetari bwe ekenyoro?* |
| **A. ACCESS TO HEALTH RESOURCES** | |
| Availability of Services  */ Obokoreri bore oo* | 1. To start can you tell me about what type of services a CHW provides to women and children?   *Ase ogochakera nonyare gontebia obokorereri mokorwa, Gochia ase abasubati na abana babo*   1. How well would you say that these services respond to women’s and children’s needs   *Mbuya ki oratebere abana naabasubati bakonyora?*   1. Are there services not provided that should be   *Inee bokoreri bonde mbore bokorwewa lakini bono botaiyio?*  If yes, what are they  *Onye nabo mborobi ore?*  Are there needs not covered in this community  *Obokoreri ki botari gotoka ase ekenyoro oreo?*  If yes, what are they  *Onye nabo mborobi obo?* |
| Challenges/Barriers */ebitango* | 1. In your opinion what are the challenges that women in your community face in accessing MNCH care in the community   *Ase amaoni ao, /mbokung’u ki abasubati na abana babo bakonyora ase ogoikera MNCH ase ebinyoro?*   1. What barriers do you see for women in attending ANC services   *Imbitaango ki okorora bikoba ebitango gochia ase abasubati bagocha ase ekiriniki?*  Delivery in a facility  *Koiborera ase nyagitari?*   1. How do you think these barriers can be   *Naki okagerete ebitango birarusigwe ?* |
| **C. BELIEFS AND PERCEPTIONS** | |
| Beliefs around MNCH Care | 1. Why do you think women do not deliver in health facilities?   *Inki okagerete okagerete abasubati batari koiborera nyagetari?*  Are there any social reasons  *Mechando ende nere eusekanete na enchera abanto bamenyete?*   1. Are there differences between younger women adolescents and older women?   *Tabauti nereo gochia ase abasubati abake abasae abasubati be emiaka ya igatwa*?  Or those that have multiple children  G*ose baria babwate abana abange?*  What are these?  *Mbiribi ebi?* |
| What are the beliefs and social norms around family planning and its use In relation to spacing of children?  */ Ebinto abanto begenete?* | 1. What are the beliefs around family planning in this community?   *Mbitoki abanto begenete ase okobanga oroiboro?*   1. As a health worker what messaging do you provide around family planning in the community?   *Aye buna omonyagitari, mbosemia ki okorwa igoro ya okobanga oroiboro?*   1. What about the beliefs around when to have children and how often?   *Inaki rend ase okonyora abana na mbwango ki?* |
|  | In your experience as a CHW what beliefs do women have around breastfeeding their newborns/ Children under 6 months  *Ase egasi yao buna omonyage, mbitoki bie egekoro begenete igoro yo okogonkia abana? abana bare inse ya emetienyi 6?* |
| FGM/ogosara abana abaiseke | 1. Can you tell me a little bit about female genital cutting in this community?   *Nonyare gokwanera ake igo igoro yo ogosara abaiseke?*  What are the reasons why this happens?  *Inki okorengereria eberete iga?*   1. As a community health worker what are some of the messages you provide around this?   *Buna omonyagetari bwe ekenyoro, mbosemia ki okorwa?* |
| **D. PRACTICES** | |
| What is the practice of men in supporting access and use of MNCH services?  *Ensemo ya abasacha nerei ase okobasira abasubati na abana babo/* (incl. accompaniment) | Now, I would like to ask about men’s roles in this community when it comes to supporting access to health services for women during pregnancy and delivery  *Bono nindigetie omanyie make ensemo ya abagaka nereri ase okobasira abasubati ase ekenyoro egere baikere.*   1. Do husbands or male family members generally accompany their wives female family members to ANC visits? Delivery? PNC?   *Abasacha nkobakoba bare abarugi babo/gose abasubati bare ase enka yabo kogenda nyagetari ekero bebwateranetie/bagochia koibora/nyuma baiboire*  Why/ *Inki*  or Why not/ *naseki etari bo?*   1. What are some of the rules of the facility around this   *Mbosemia ki bokorwewa gochia ase aya?*   1. Are men encouraged to play this role   *Abasacha ngosegetwa bare gokora aya?*  If yes, how  *Onye nabo ngecho ki?*  If no, why  *Onye taribo, naseki?*   1. What would prevent men from playing an active role in supporting women’s health issues   *Ninki gekobatanga abasacha gotabasira abasubati ase amangana anyagetari?* |
| Health Worker Treatment  /*Omogwenia inaki akoirwa* | 1. In your experience, how do male clients treat you   *Ase oborori bwao ingaki abasacha bagokiira?* |
| Exclusive Breastfeeding/ *okomogopngia omwana amabere angina* | 1. What are the customs around breastfeeding in this community   *Mbiribi egekoro kegoteba ase okogongia omwana amabere angina?*   1. When a woman delivers in the facility what is she instructed to do in terms of feeding her newborn   *Ekero omongina akoiborera nyagitari inki agosemigwa gokora ase engecho yo okomorageria omwana oye?* |
| **E. POLICIES, RULES** | |
| Health Facility Governance/*oborai bwe enyagetari* | 1. What are the tasks performed by male and female CHWs   *Mbiribi bigokorwa na omonyagetari omsacha gose omokungu bwe ekenyoro?*  Is there a difference between those performed by men and those by women  *Mbiroo biria bigokorwana abakungu na abasacha kando?* |

**Consent Form KII/FGD:**

| **AGA KHAN FOUNDATION, EAST AFRICA//** **AGA KHAN UNIVERSITY (EAST AFRICA)/AGA KHAN HEALTH SERVICES** |
| --- |
|  |
| **Access to Quality Care through Extending and Strengthening Health Systems (AQCESS): Gender assessment of maternal, neonatal and child health indicators in Kaloleni and Bomachoge-Borabu sub-counties in Kenya.** |
|  |
|  |

Hello, my name is ________________________________________________ and I am working with the Aga Khan Development network who have partnered with the Ministry of Health of the government of Kenya to conduct a Gender assessment study at the community level for maternal, neonatal and child indicators of this community in Kaloleni, Rabai and Bomachoge subcounty [adopt as necessary]. I am going to give you information and request you to be part of this survey. We would very much appreciate your participation.

**Purpose**

The overall objective of the Gender assessment is intended to provide AQCESS project staff with detailed information about the key gender issues within the context of the two project sub-county implementation areas of Kaloleni and Kisii, and related to the project interventions. Specifically, a focus on gender equality examines how differences in power relations result in differential risks, vulnerabilities, and outcomes in health for men and women

**Participant selection**

To take part in the current assessment you must be a resident of Kaloleni, Rabai and Bomachoge Borabu sub-counties. You have been selected as a respondent because fit in the selection criteria.

**Procedures**

The discussion/interview will take about one hour and will include questions about your household, your health and the health of your children under 5 years of age.

**Risks and discomforts**

The interview has no risks to you or your health. However, if a question causes any anxiety or discomfort you may choose not to answer without giving a reason and we will proceed to the next question.

**Benefits**

The findings from the Gender assessment for the AQCESS project which will be based on your participation will inform the activities and interventions that we plan to implement in this area. These activities are aimed at improving the services given by the subcounty department of health to Mothers, newborns and children under the age of 5 years in order to improve their health.

**Confidentiality**

All information collected from the survey will be kept confidential. The completed survey and signed Consent Form will be placed together. All research materials will be given a confidential number that will be known only by a few people involved in this research. Results of this study will not be released or reported in any way that might allow for individual participants to be known by people not involved in the study. All research materials will be assigned a confidential number for coding purposes. Results will not be released or reported in any way that might allow for identification of individual participants. All information will be aggregated and will be used only for community reference and not for individuals. Your name will not be associated with the findings.

**Voluntary Participation**

All participation in this research is voluntary. You are free to decide if you want to take part or not. If you do agree to take part now, you can change your mind at any time during the interview without any implications. But we hope you will participate to the end.

**Contact information**

For more information about this study, you can contact the researchers who are responsible: Ms.Angela Ngetich (+254 780660080) or Lucy Nyaga (041 2226950), Kennedy Mulama ( 0726741499 )

**Consent Statement**

I have read the preceding information, or it has been read to me. I have had the opportunity to ask questions about it and they have been answered to my satisfaction. I consent voluntarily to be a participant in this study and understand that I have the right to withdraw from the interview/discussion at any time.

**🞎** I agree to be interviewed (*please tick*) **🞎** I do not agree to be interviewed (*please tick*)

**Respondents Signature:** ______________________________ **Date:** _____________________

**Thumbprint of the respondent named above if they cannot write:** _____________________

I, the undersigned, have fully explained the relevant details of this survey to the respondent to consent.

**Enumerator’s name:** ___________________________________________________________

**Enumerator’s signature:** _____________________________ **Date:** _____________________

**Consent Form interpreted in Gisii:**

| **EUNIBASITI YA AGA KHAN (ABIRIKA YA MOCHA)/OBOROSO BWA AGA KHAN, ABIRIKA YA MOCHA/OBOKORERI NA OBOGWENIA BWA AGA KHAN** | |
| --- | --- |
|  | |
| **Ogoikera oborendi na gokongia obogwenia (AQCESS):Esabei ya chinyagetari na Ebinyoro erengete abangina bebwateranetie gose ekero bakonyora abana,ebingwerere biabo na abana ime ase ebinyoro bia Bomachoge Borabu.** |  |
|  |  |
|  |  |

Imbuya ore?,ase amarieta , ________________________________________________na nigo inkokora egasi na Ekeombe kia Aga Khan Development network ekio gekobwaterana na ewisara ya obochenu aiga Kenya gokora obotuki ase ebinyoro biaito goro ya abangina,ebingwerere na abana abake erio egere korora ebinto biria bire omochando ase bareaiga Bomachoge Borabu.Ningoe amangana make na ningake buya mono oise gwancha gosoa ase ebigambo ebi .

**Eganga**

Eganga enene ya amaboria aya ase chinyomba nokogania komanya kiafia aba iga ingaki ere;Abasubati bare emiaka gati ya 15-49,Ebingwerere bire inse ya omwaka oyomo(1yr),na abane abake bare inse ya emiaka etano ase chinka gete engeigo chirachorwe gosoa ase amaboria aya. Amaoni onsi torasangererie natoe amangana ekeombe gia AQCESS keratumie gotoraa nga ingaki torakongie na gotingia oboweniwa seino igaiga, okogania okonene koragete nga ingaki amakweri akobanyora abangina bebwateranetie,Ebingwerere na abana bare inse ya emiaka etano.

**INGAKI ORACHORWE KOBORIGWAA BWA AMABORIA AYA**

Oyo orachorwe koborigwa amaboria aria aya goika abe omomenyi obo Bomachoge Borabu sub-counti.Enka yao yachorirwe yokorabaraba aiga naria ase engencho ya amaboria aya. Aye nigo gwachorirwe ase engecho nigo ore omosubati bwa emiaka ebwenerete ase enka eye naende nomonyete buya mono abanto bonsi bamenyete ase enka eye .Goetania igo nigo torigetie kobabori amoboria make arengete abasubati be emiaka gati ya 15-49 (years) abwo banyarire gose baibore abana ebingwerere ase emiaka etato yaetire (nayenabo okoba oyomo obo onye kore ase abwo bakwanirwe), na negenire inga nobe oyobwo ogokonya okonene.

**Omoroberio**

Amangana toragambe na amaboria aya iga nigo araire ange ensa eyemo na chitageka emerongo ebere na nigo arabwatane na amaboria ya Enka yao, afia yao neria ya abana bao bare inse ya emiaka etano.

**Okobeka obogima ase akongu gose goteigwa inga imbuya**

Amaboria aya tabwati kende kerasumbue omobere oo kiafia. Korende koranyore akoreteire bokongu gete ase omobere nobwate obosibore bwo gotairaneria otarweti esababu ende yonsi naigo nigo toragenderere na amaboria akobwatia.

**OBUYA**

Amachibu ya amaboria aya korwa ase ekeombe gia iga AQCESS nigo mono arategemee okwerwa kwao ayio arairere ogokorwa kwa ebinto gete bigochia gokorwa ase ebinyoro biaito bia Bomachoge Borabu project. Ebinto ebi iga bigochia gokorwa nigo birengete goonchoreria obokoreri bwa ewisara ya obochenu ase ekerongo kebwenerete gochia ase amagima yaabangina bonsi na abana babo ebingwerere, nabaria bare inse ya emiaka etano.

**Kobeka Obobisi**

Amachibu onsi korwa ase amaboria aya nigo arabekwebobisi mono.Amasakara aya abekire esei yo ogoanchera amaboria nigo arabekwe amo na amachibu ya amaboria.Amasakara onsi ya amaboria nigo araegwe enamba ya bobisi eyio eramanyekane na abanto abake sana bausekanete na amangana aya. Nchera ende teri amachibu ya amaboria aya aranekerwe goikera ekerengo kia oyoirete ensemo ya okoiraneria aramanyekane na abanto batabwati ensemo bagokora ase amangana aya. Amasakara onsi abwate amachibu aya naegwe enamba ya bobisi ase engencho yo okoenana chinamba gete chia bene ekero araganerigwe gotumeka.Amachibu onsi nigo arabangwe na nigo aratumeke na ekenyoro gionsi na tari ase omonto gete bweka bene. Rieta riao tirigotwara boamate bonde na amachibu.

**Okwerwa Koiraneria**

Ogosoa ase amaboria aye nigo kore okwerwa. Nore na obosibore bwo ogochora gose nkoira ore ensemo gose yaya. Onyore gwancheranire koira ensemo bono, nabo gwancheire gochenchia ebirengererio biao insa chinde chionsi bwoba bonde botaiyio. Korende neganerie nkogenderera goika omoerio.

**CHINAMBA CHIA BARIA ORAIKERE KONYORA AMANGANA**

Erio egere konyora obwaagare igoro ya amangana aya nabo okobaikera aba iga. For more information about this study, you can contact the researchers who are responsible: Dr. Michaela Mantel (020 3662958). Mr. Felix Agoi (041 2226950) or Lucy Nyaga (041 2226950), Jane Wanyama (0572022244).

**OGWANCHERANA**

Nasomire amangana aya iga, gose nasomeirwe. Inanyora ribaga riokoboria amaboria na koiranerigwa goikera ekerengo kio ogoisanekigwa ebirengererio biane kobwatekana na amaboria aya. Nancheranire kwerwa gosoa ase amaboria/ebigambero aya/ebi naende namanyire inga nimbwate obosibore bwo ogotigera inchera ngaki chinde yonsi.

**🞎Nancheranire koborigwa (**beka etiki) **🞎 Tindi tayare koborigwa**  (beka etiki)

**Esei ya oyokoiraneri:** _______________________**Chitariki : _____________________**

**Oyokoiraneria abeke egechara onye tamanyeti korika na gosoma**

Nche oyo ngochia koboria amaboria aya iga nagareirie ase obotambe aria onsi are ime ase amaboria aya iga gochia ase oyotakeire koyairaneria egere anche koiraneria.

**Erieta rio oyokoboria:** ___________________________________________________________

**Esei ya oria ogochia koboria:** _____________________**Chitariki** **:** _____________________
