# Supplementary material for: Deliver on Your Own: Disrespectful Maternity Care in rural Kenya
Source: PLoS One. 2020 Jan 7;15(1):e0214836. doi: 10.1371/journal.pone.0214836 (PMC6946164; doi:10.1371/journal.pone.0214836)
Supplement: S3 Appendix — (DOCX) [file pone.0214836.s003.docx]

| **Appendix 3. Barriers to Skilled Delivery Service in Bomachoge-Borabu Kisii** | | | | | | | | | | | | | | | | | | | | | | | |
| --- | --- | --- | --- | --- | --- | --- | --- | --- | --- | --- | --- | --- | --- | --- | --- | --- | --- | --- | --- | --- | --- | --- | --- |
|  | | ***Key informant interviews*** | | | | | | | | | | | | | ***FGDs*** | | | | | | | | |
| ***Sessions*** | | **1** | **2** | **3** | **4** | **5** | **6** | **7** | **8** | **9** | **10** | **11** | | **12** | **13** | **14** | **15** | **16** | **17** | **18** | **19** | **20** | **Count** |
|  | |  |  |  |  |  |  |  |  |  |  |  | |  |  |  |  |  |  |  |  |  |  |
| Health worker bad attitude/ harassment/ abuse of mothers | |  |  |  |  | x | x |  |  |  |  | x | |  | x | x | x |  | x | x | x | x | 10 |
| Distance | | x | x |  |  |  |  |  |  |  | x |  | |  | x | x | x |  | x |  | x |  | 8 |
| Mothers requested to buy delivery commodities | |  |  |  |  |  | x |  | x |  | x |  | |  |  | x |  |  | x | x |  | x | 7 |
| Health worker manipulation of clients/ staff corruption/ dishonesty/ favoritism | |  |  |  |  |  | x |  |  |  |  |  | |  | x |  | x | x |  | x |  |  | 5 |
| Unqualified / incompetent / student health care workers | |  |  |  |  |  |  |  |  |  |  |  | |  |  |  | x | x |  | x |  | x | 4 |
| Lack of essential supplies | |  |  |  |  |  |  |  |  |  |  |  | |  | x | x |  | x |  |  |  |  | 3 |
| Patient flow / long queues/ slow service/ lay out of related service points | |  |  |  |  |  |  |  |  |  |  |  | |  |  |  |  |  |  | x | x | x | 3 |
| Health worker strikes | |  |  |  |  |  |  |  |  | x |  |  | |  |  |  |  |  |  |  |  | x | 2 |
| No/limited service at night & during the weekend | |  |  |  |  |  |  |  |  |  |  |  | |  |  |  |  |  |  | x | x |  | 2 |
| ANC Clinic card and stamp requirement | | x |  |  |  |  |  |  |  |  |  |  | |  |  |  |  |  |  | x | x |  | 2 |
| Exercises required of women during labor | |  |  |  |  |  |  |  |  |  |  |  | |  |  |  |  |  |  | x |  |  | 1 |
| Rumors on stealing/ exchange of babies with dead fetus | |  |  |  |  |  |  |  |  |  |  |  | |  |  | x |  |  |  |  |  |  | 1 |
| HCW failure to disclose patient’s diagnosis & associated treatment without patient consent | |  |  |  |  |  |  |  |  |  |  |  | |  |  |  |  |  |  | x |  |  | 1 |
| Politics and poor facility management especially in FBO | |  |  |  |  |  |  |  |  |  |  |  | |  |  |  |  |  |  | x |  |  | 1 |
| Lack of adolescent support services | |  | x |  |  |  |  |  |  |  |  |  | |  |  |  |  |  |  |  |  |  | 1 |
| Lack of proper documentation at health facility | |  |  |  |  | x |  |  |  |  |  |  | |  |  |  |  |  |  |  |  |  | 1 |
| Accompaniers not allowed to assist the mother in the ward | |  |  |  |  |  |  |  |  |  |  |  | |  |  |  |  | x |  |  |  |  | 1 |
| Client asked to fuel health facility vehicles/ ambulance when referred | |  |  |  |  |  |  |  |  |  |  |  | |  |  |  |  | x |  |  |  |  | 1 |
| Lack of food for mothers at maternity | |  |  |  |  | x |  |  |  |  |  |  | |  |  |  |  |  |  |  |  |  | 1 |
| ***Individual and community level factors*** | | | | | | | | | | | | | | | | | | | | | | | |
| Lack transport fare and means | | x |  |  |  |  | x |  |  | x | x | x | | x |  | x |  | x | x | x | x |  | 11 |
| Social and self-Stigma (e.g. young mothers) | | x | x |  |  | x | x |  |  |  |  |  | |  |  | x |  |  |  | x |  | x | 7 |
| Lack of support and permission from spouse | |  |  |  |  |  | x |  |  |  | x |  | |  |  |  |  | x |  |  |  | x | 4 |
| Lack of accompanier | | x | x |  |  |  |  |  |  |  |  |  | |  |  |  |  | x |  |  |  |  | 3 |
| Fear of HF episiotomy, caesarian birth and HIV test | |  |  |  |  |  |  |  |  | x |  |  | |  |  | x |  |  |  |  |  | x | 3 |
| Ignorance about skilled delivery | |  |  |  |  |  |  |  |  |  | x |  | |  |  |  |  |  |  |  |  |  | 1 |
| Marital/ family conflicts | | x |  |  |  |  |  |  |  |  |  |  | |  |  |  |  |  |  |  |  |  | 1 |
| Religious support for home birth | |  |  |  |  |  |  |  |  |  |  |  | |  |  | x |  |  |  |  |  |  | 1 |
| **Key** |  | |  |  | | | | | | | |  |  |  |  |  |  |  |  |  |  |  |  |
| **1** | HF Provider 1 | | **13** | Women <19yrs 1 (rural, >5km) | | | | | | | |  |  |  |  |  |  |  |  |  |  |  |  |
| **2** | HF Provider 2 | | **14** | Women <19yrs 2 (urban, <5km) | | | | | | | |  |  |  |  |  |  |  |  |  |  |  |  |
| **3** | Local govt leader 1 | | **15** | Women >20yrs 1 (rural, >5km) | | | | | | | |  |  |  |  |  |  |  |  |  |  |  |  |
| **4** | Local govt leader 2 | | **16** | Women >20yrs 2 (urban, <5km) | | | | | | | |  |  |  |  |  |  |  |  |  |  |  |  |
| **5** | MoH Representative 1 | | **17** | Male 1 (rural, >5km) | | | | | | | |  |  |  |  |  |  |  |  |  |  |  |  |
| **6** | MoH Representative 2 | | **18** | Male 2 (urban, <5km) | | | | | | | |  |  |  |  |  |  |  |  |  |  |  |  |
| **7** | Religious leader 1 | | **19** | CHC 1 (rural, >5km) | | | | | | | |  |  |  |  |  |  |  |  |  |  |  |  |
| **8** | Religious leader 2 | | **20** | CHC 2 (urban, <5km) | | | | | | | |  |  |  |  |  |  |  |  |  |  |  |  |
| **9** | CHV 1 | |  |  | | | | | | | |  |  |  |  |  |  |  |  |  |  |  |  |
| **10** | CHV 2 | |  |  | | | | | | | |  |  |  |  |  |  |  |  |  |  |  |  |
| **11** | Women leader 1 | |  |  | | | | | | | |  |  |  |  |  |  |  |  |  |  |  |  |
| **12** | Women leader 2 | |  |  | | | | | | | |  |  |  |  |  |  |  |  |  |  |  |  |
